# Autophagy dysregulation via the USP20-ULK1 axis in the HERC2-related neurodevelopmental disorder

**DOI:** 10.1038/s41420-024-01931-6

**Published:** 2024-04-03

**Authors:** Joan Sala-Gaston, Eva M. Pérez-Villegas, José A. Armengol, Lettie E. Rawlins, Emma L. Baple, Andrew H. Crosby, Francesc Ventura, Jose Luis Rosa

**Affiliations:** 1 Department of Physiological Sciences, University of Barcelona (UB), Bellvitge Biomedical Research Institute (IDIBELL), L’Hospitalet de Llobregat, Spain; 2 Department of Physiology, Anatomy and Cell Biology, University Pablo de Olavide, 41013 Seville, Spain; 3 RILD Wellcome Wolfson Medical Research Centre, RD&E (Wonford) NHS Foundation Trust, University of Exeter Medical School, Exeter, UK; 4 Peninsula Clinical Genetics Service, Royal Devon & Exeter Hospital (Heavitree), Exeter, UK

**Keywords:** Macroautophagy, Autism spectrum disorders, Mechanisms of disease

## Abstract

Sequence variants in the *HERC2* gene are associated with a significant reduction in HERC2 protein levels and cause a neurodevelopmental disorder known as the HERC2-related disorder, which shares clinical features with Angelman syndrome, including global developmental delay, intellectual disability, autism, and movement disorders. Remarkably, the *HERC2* gene is commonly deleted in individuals with Angelman syndrome, suggesting a potential contribution of HERC2 to the pathophysiology of this disease. Given the known critical role of autophagy in brain development and its implication in neurodevelopmental diseases, we undertook different experimental approaches to monitor autophagy in fibroblasts derived from individuals affected by the HERC2-related disorder. Our findings reveal alterations in the levels of the autophagy-related protein LC3. Furthermore, experiments with lysosomal inhibitors provide confirmation of an upregulation of the autophagy pathway in these patient-derived cells. Mechanistically, we corroborate an interaction between HERC2 and the deubiquitylating enzyme USP20; and demonstrate that HERC2 deficiency leads to increased USP20 protein levels. Notably, USP20 upregulation correlates with enhanced stability of the autophagy initiating kinase ULK1, highlighting the role of HERC2 as an autophagy regulator factor through the USP20-ULK1 axis. Moreover, we show that p38 acts as a modulator of this pathway, since p38 activation disrupts HERC2-USP20 interaction, leading to increased USP20 and LC3-II protein levels. Together, these findings uncover a previously unknown role for HERC2 in autophagy regulation and provide insights into the pathomolecular mechanisms underlying the HERC2-related disorder and Angelman syndrome.

## Introduction

Macroautophagy, hereafter referred to as autophagy, is characterized by the engulfment of cellular material by a double-membrane structure called phagophore, which eventually seals forming the autophagosome. Mature autophagosomes are transported along microtubules toward lysosomes, fusing with them and resulting in the degradation of the cargo [1, 2]. Signals that activate the initiation of autophagy usually originate from various stress conditions such as starvation, oxidative stress, protein aggregation, hypoxia, and others. Typically, most signaling cascades converge in the activation of the autophagy initiating kinase ULK1 [3]. During phagophore elongation, the cytosolic form of the LC3 protein (LC3-I) is conjugated to a phosphatidylethanolamine (PE), forming LC3-II, which is incorporated to the autophagosome membranes [4]. Several genetic studies have highlighted the important role of autophagy in human brain development, regulating important neurodevelopmental processes such as cell proliferation of neuronal precursors, axon outgrowth, and synaptic formation [5]. Consequently, dysregulation of autophagy is associated with a wide range of neurodevelopmental disorders, including agenesis of the corpus callosum, macrocephaly, autism, intellectual delay, and paraplegias [6]. Furthermore, autophagy has emerged as a significant process in the pathogenesis of several neurodegenerative diseases including Alzheimer’s, Huntington’s, and Parkinson’s diseases [7].

HERC2 is a ubiquitin ligase involved in various physiological processes, including membrane trafficking, inflammation, immune response, DNA repair, and the cellular stress response [8–11]. In situ hybridization studies in mice have provided evidence of *Herc2* expression in different areas of the nervous system such as the isocortex, hippocampus, thalamus, hypothalamus, midbrain, pons, medulla and cerebellum [12]. The *HERC2* gene is located near an imprinting region of chromosome 15 associated with neurodevelopmental disorders like Angelman syndrome. Notably, the *HERC2* gene is commonly deleted in Angelman syndrome patients [13–15]. Moreover, biallelic hypomorphic variants of *HERC2* are linked with a neurodevelopmental disorder known as the HERC2-related disorder, which clinically resembles Angelman syndrome and is characterized by global developmental delay, intellectual disability, autism, and movement disorders (OMIM # 615516) [16–20]. Collectively, these observations show the important role of HERC2 in neurodevelopment and suggest a potential contribution of HERC2 to the pathophysiology of Angelman syndrome.

Studies conducted in mice suggested an association between HERC2 and the autophagy pathway. Knockout mice lacking *Herc2* are not viable, showing that *Herc2* is essential for embryonic development. However, mice with a targeted inactivation of one *Herc2* allele, named the *Herc2*^*+/530*^ mice, exhibit a reduction in Herc2 protein levels and display motor coordination dysfunction. Morphological analysis of the cerebellum in these animals revealed degeneration of Purkinje cells, with signs of increased accumulation of autophagosomes and lysosomes [21]. These findings suggested a potential dysregulation of autophagy, although the direct involvement and precise role of Herc2 in autophagy was not elucidated.

In this study, we analyzed patient-derived fibroblasts from individuals with the HERC2-related disorder harboring the homozygous HERC2 p.Pro594Leu variant (HERC2 P594L). Our investigation unveils a novel role for HERC2 as an autophagy regulator factor through the USP20-ULK1 axis, providing new insights into the pathomolecular basis of HERC2-related disorder and Angelman syndrome.

## Results

### Patient-derived cells with a homozygous mutation in human *HERC2* gene show autophagy dysregulation

**Fig. 1:**
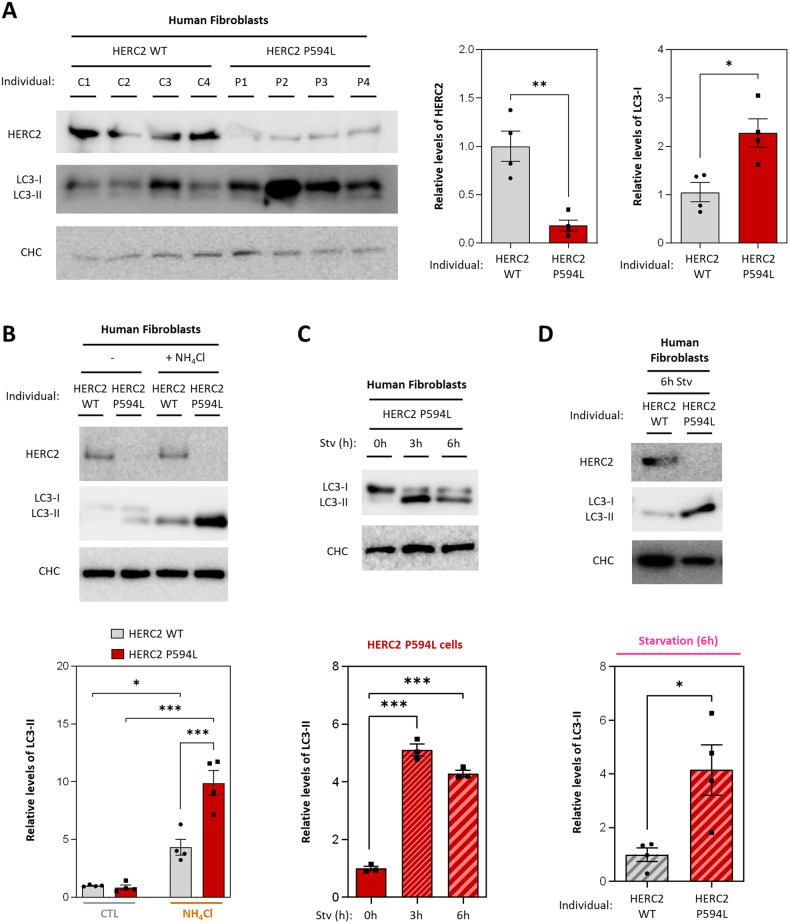
Patient-derived cells with a homozygous mutation in human *HERC2* gene show autophagy dysregulation. **A** Immunoblot analysis was performed on lysates derived from human skin fibroblasts, involving samples from four control individuals with the wild-type HERC2 (C1, C2, C3 and C4) and four patients with the HERC2 P594L mutant variant (P1, P2, P3 and P4). LC3 was detected using an anti-LC3B antibody. Band intensities corresponding to HERC2 and LC3-I were quantified and subsequently normalized using clathrin heavy chain (CHC) protein levels as a loading control. **B** HERC2 WT and HERC2 P594L cells were subjected to a 4-h treatment with 20 mM NH_4_Cl when indicated. LC3 was detected using an anti-LC3B antibody. Levels of LC3-II were quantified and subsequently normalized using clathrin heavy chain (CHC) protein levels as a loading control. **C** HERC2 P594L cells were subjected to Earle’s Balanced Salt Solution (EBSS)-induced starvation (Stv) for 3 or 6 h or left in DMEM complete medium as the untreated condition (0 h). Levels of LC3-II were detected and quantified as in (**B**). **D** HERC2 WT and HERC2 P594L cells were subjected to EBSS-induced starvation for 6 h. Levels of LC3-II were detected and quantified as described in (**B**). The results are presented as fold changes relative to the control condition. Plots display the mean ± standard error of the mean (SEM). Representative results are shown for experiments that were independently repeated at least three times, and individual data points for each independent experimental repetition are represented as individual dots on the graphs. Significance was determined using unpaired Student’s t-test in (**A**) and (**D**); one-way analysis of variance (ANOVA) with Tukey’s correction for multiple comparisons in (**B**); and two-way ANOVA with Tukey’s correction for multiple comparisons in (**C**). Significance levels: *p < 0.05; **p < 0.01; ***p < 0.001.

Dysregulation of autophagy is implicated in various neurodegenerative and neurodevelopmental conditions [6, 7]. Thus, we wondered whether autophagy might be deregulated in cells derived from individuals harboring the HERC2 P594L variant associated with the HERC2-related disorder. To explore this, we assessed protein levels of the autophagosome marker LC3 using an anti-LC3B antibody. Cells carrying the HERC2 P594L variant displayed reduced HERC2 protein levels (Fig. 1A). We employed an anti-HERC2 antibody which was raised against a peptide consisting of amino acid residues 1781 to 1974 of HERC2 as the immunogen. As such, this commercial antibody recognizes a common region for both HERC2 wild-type (WT) and the P594L variant [17]. Interestingly, HERC2 P594L cells exhibited increased protein levels of LC3-I. However, the levels of lipidated LC3 (LC3-II) could not be properly analyzed in this model due to low expression under basal conditions (Fig. 1A). These findings suggested that HERC2 deficiency affects LC3 protein levels, which may be indicative of a dysregulation of autophagy.

To determine whether and how the process of autophagy is dysregulated, it is essential to analyze the levels of LC3-II, as this lipidated form is incorporated into nascent phagophores during the autophagic process. To investigate this, cells were treated with the lysosomal inhibitor NH_4_Cl for 4 h. The treatment with NH_4_Cl effectively inhibited autophagolysosome degradation in both HERC2 WT and HERC2 P594L cells, as indicated by the accumulation of LC3-II protein (Fig. 1B, lanes 1 and 2 compared with lanes 3 and 4, respectively). Moreover, under lysosomal inhibition, HERC2 P594L cells exhibited higher LC3-II protein levels than HERC2 WT cells (Fig. 1B, lane 3 compared with lane 4). It is noteworthy that if autophagy were blocked in patient-derived cells, LC3-II levels would not increase after lysosomal blockade. To further validate these findings, we transferred HERC2 P594L cells to a starvation medium (EBSS) for 3 and 6 h and analyzed LC3 conversion. The results revealed that conversion of LC3-I to LC3-II occurred, and elevated LC3-II protein levels were detected after both 3 and 6 h of starvation, providing compelling evidence that HERC2-deficient cells can indeed initiate the autophagic pathway (Fig. 1C). Subsequently, we compared LC3-II protein levels between HERC2 WT and HERC2 P594L cells under conditions of starvation, revealing higher LC3-II protein levels in HERC2 P594L cells (Fig. 1D). Collectively, these data showed that reduced levels of HERC2 protein induces autophagy.

### HERC2 deficiency enhances the autophagy pathway

**Fig. 2:**
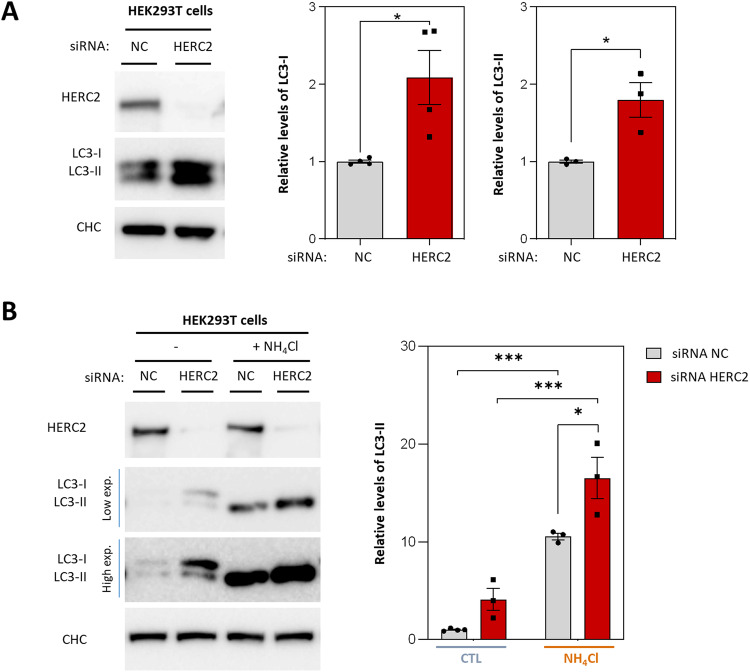
HERC2 deficiency enhances the autophagy pathway. **A** HEK293T cells were transfected with either a negative control siRNA (NC) or a siRNA targeting HERC2. Subsequently, the expression levels of the indicated proteins were assessed by immunoblot analysis. LC3 was detected using an anti-LC3B antibody. Quantification of LC3-I and LC3-II proteins was performed and the data were normalized using clathrin heavy chain (CHC) protein levels as an internal loading control. **B** Negative control (NC) or HERC2 knocked-down cells were treated with 20 mM NH_4_Cl for 4 h as indicated. Levels of LC3-II were detected and quantified as in (**A**). The results are presented as fold changes relative to the control condition. Plots display the mean ± standard error of the mean (SEM). Representative results are shown for experiments that were independently repeated at least three times, and individual data points for each independent experimental repetition are represented as individual dots on the graphs. Significance was determined using unpaired Student’s t-test in (**A**) and two-way ANOVA with Tukey’s correction for multiple comparisons in (**B**). Significance levels: *p < 0.05; ***p < 0.001.

To validate that the observed autophagy dysregulation in patient-derived cells was primarily attributable to the reduction of HERC2 protein levels, we conducted knockdown experiments in the HEK293T cell line. Notably, HERC2 knockdown resulted in a significant increase in both LC3-I and LC3-II protein levels (Fig. 2A). Then, as previously done with human fibroblasts, we subjected HEK293T cells to a 4-h treatment with NH_4_Cl. This treatment effectively induced the accumulation of LC3-II in both control and HERC2 knocked-down cells (Fig. 2B, lanes 1 and 2 compared with lanes 3 and 4, respectively). Moreover, under conditions of lysosomal inhibition, HERC2 knocked-down cells exhibited higher LC3-II protein levels compared to control cells (Fig. 2B, lane 3 compared with lane 4). These results strongly suggested that HERC2 depletion induces autophagy, thereby validating the prior observations made in patient-derived cells.

### HERC2 regulates protein levels of the USP20-ULK1 axis

**Fig. 3:**
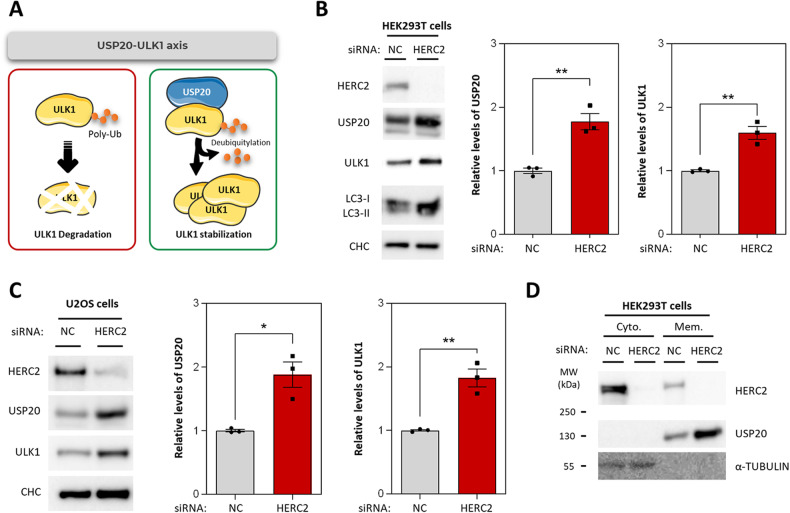
HERC2 regulates protein levels of the USP20-ULK1 axis. **A** Schematic representation of the USP20-ULK1 axis. ULK1 protein levels are regulated post-translationally by ubiquitylation-dependent protein degradation (left panel). The deubiquitylating enzyme USP20 interacts with ULK1, catalyzing its deubiquitylation and thereby enhancing ULK1 stability (right panel). HEK293T cells (**B**) or U2OS (**C**) were transfected with either a negative control siRNA (NC) or a siRNA targeting HERC2. Subsequently, the expression levels of the indicated proteins were assessed by immunoblot analysis. The intensity bands corresponding to USP20 and ULK1 were quantified and subsequently normalized using clathrin heavy chain (CHC) protein levels as a loading control. The results are presented as fold changes relative to the control condition. Plots display the mean ± standard error of the mean (SEM). Representative results are shown for experiments that were independently repeated at least three times, and individual data points for each independent experimental repetition are represented as individual dots on the graphs. Significance was determined using unpaired Student’s t-test. Significance levels: *p < 0.05; **p < 0.01. **D** Control cells and HERC2 knocked-down cells were fractionated into cytosol (cyto.) and membrane (mem.) using 0.015% digitonin. Immunoblotting of α-TUBULIN (cytosolic control) shows the successful separation of the cytosol and membrane. Data are representative of two independent experiments.

Previous studies have demonstrated that USP20 catalyzes the deubiquitylation and subsequent stabilization of ULK1, resulting in the facilitation of autophagy initiation [22] (Fig. 3A). On the other hand, HERC2 has been shown to modulate the stability of USP20 by promoting its ubiquitylation-mediated proteasomal degradation [23, 24]. However, to date, no direct connection between HERC2, the USP20-ULK1 axis, and the regulation of autophagy has been reported. To address this knowledge gap, we examined the protein levels of USP20 and ULK1 after HERC2 knockdown in HEK293T cells. Our finding revealed a significant increase in the protein levels of both USP20 and ULK1 in HERC2 knocked-down cells (Fig. 3B). The same effect was observed in the U2OS cell line (Fig. 3C).

Since USP20 is known to be localized to the cell membrane [25], we conducted experiments to determine if its upregulation following HERC2 depletion influences its subcellular localization. As expected, we detected USP20 in the membrane fraction of control cells. Interestingly, HERC2 was present in both the cytosolic and membrane fractions. Notably, the subcellular localization of USP20 remained unaltered upon HERC2 silencing, indicating that the observed USP20 upregulation primarily occurs within the membrane fraction (Fig. 3D).

### USP20 controls ULK1 protein stability and regulates autophagy

**Fig. 4:**
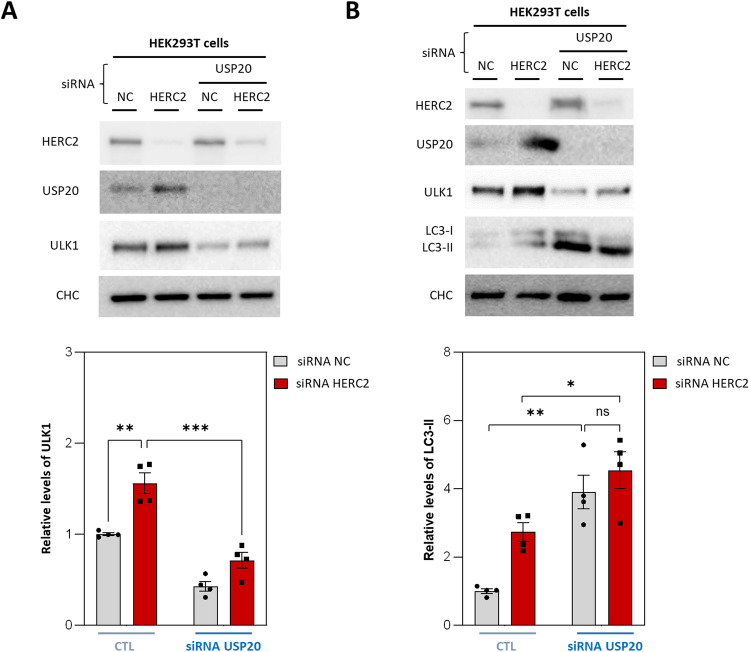
USP20 controls ULK1 stability and regulates autophagy. **A**, **B** HEK293T cells were transfected with either the NC or HERC2 siRNA. A siRNA against USP20 was added under the indicated conditions, and the specified protein levels were analyzed by immunoblot. LC3 was detected using an anti-LC3B antibody. The intensity bands corresponding to ULK1 (**A**) and to LC3-II (**B**) were quantified and subsequently normalized using clathrin heavy chain (CHC) protein levels as a loading control. The results are presented as fold changes relative to the control condition. Plots display the mean ± standard error of the mean (SEM). Representative results are shown for experiments that were independently repeated at least three times, and individual data points for each independent experimental repetition are represented as individual dots on the graphs. Significance was determined using two-way ANOVA with Tukey’s correction for multiple comparisons. Significance levels: ns no significance; *p < 0.05; **p < 0.01; ***p < 0.001.

To confirm whether the increase in ULK1 protein levels observed following HERC2 silencing is a consequence of the upregulation of USP20, we knocked-down USP20 in HERC2-silenced cells. USP20 knockdown significantly attenuated the rise in ULK1 protein levels observed after HERC2 depletion (Fig. 4A). Next, we analyzed LC3-II protein levels in those cells. Interestingly, the single knockdown of USP20 triggered an increased accumulation of LC3-II (Fig. 4B, lane 1 compared with lane 3). Notably, the double knockdown of USP20 and HERC2 did not lead to a further increase (Fig. 4B, lane 3 compared with lane 4). The abolishment of differences in LC3-II protein levels between control and HERC2-silenced cells under USP20 knockdown strongly suggested that the effects observed in the autophagy pathway as a result of HERC2 deficiency depend on USP20.

### Activation of p38 drives upregulation of LC3-II and USP20

**Fig. 5:**
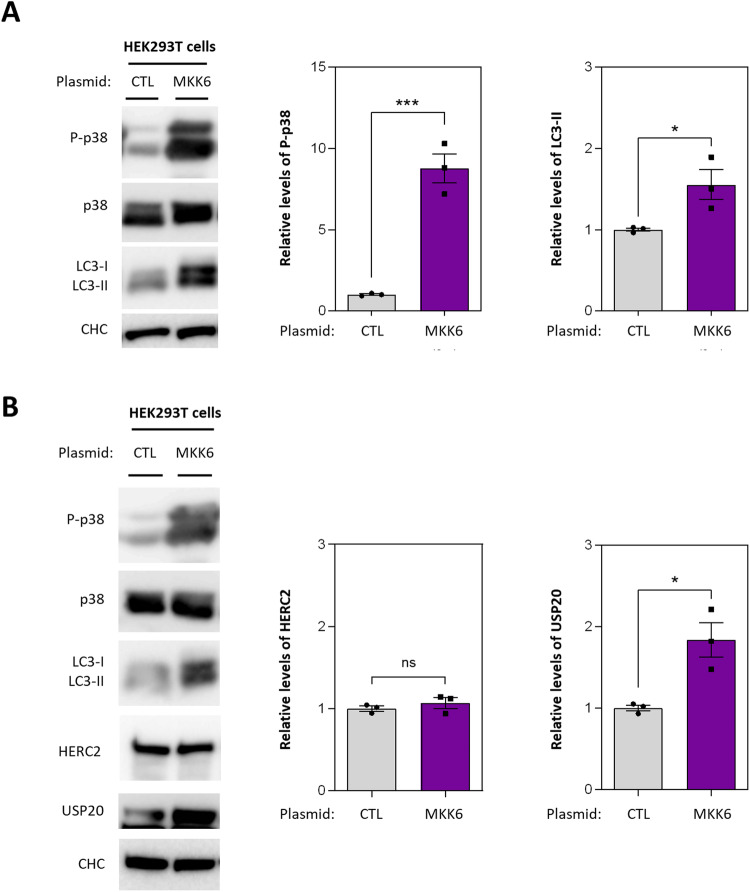
Activation of p38 drives upregulation of LC3-II and USP20. **A**, **B** HEK293T were transfected with either a control plasmid (CTL) or a plasmid encoding a constitutively active MKK6 protein (MKK6). Cell lysates were analyzed by immunoblot using the indicated antibodies. LC3 was detected using an anti-LC3B antibody. Phospho-p38 (P-p38) and LC3-II protein levels were quantified and normalized based on total p38 protein levels or CHC protein levels, respectively, in (**A**). Intensity bands corresponding to HERC2 and USP20 were quantified and normalized based on CHC protein levels in (**B**). The results are presented as fold changes relative to the control condition. Plots display the mean ± standard error of the mean (SEM). Representative results are shown for experiments that were independently repeated at least three times, and individual data points for each independent experimental repetition are represented as individual dots on the graphs. Significance was determined using unpaired Student’s t-test. Significance levels: ns = no significance; *p < 0.05; ***p < 0.001.

HERC2 modulates p38 phosphorylation by controlling C-RAF ubiquitylation-mediated proteasomal degradation [26]. Furthermore, p38 can act as a positive regulator of autophagosome formation [27]. Building upon this background, we investigated whether p38 signaling is involved in the HERC2-dependent autophagy. We initially transfected HEK293T cells with a plasmid encoding a constitutively active kinase MKK6, which leads to the phosphorylation and activation of p38. As anticipated, transfection of MKK6 effectively induced p38 phosphorylation and incremented protein levels of LC3-II (Fig. 5A). These findings showed that p38 activation stimulates autophagosome formation and promotes the autophagy pathway in HEK293T cells.

Next, we examined whether there were any alterations in the protein levels of HERC2 or USP20. Interestingly, no changes in HERC2 protein levels were observed in cells transfected with MKK6. However, we observed a notable upregulation of USP20 (Fig. 5B). In summary, these findings suggest that p38 activation plays a regulatory role in autophagy, involving USP20 as a component of the molecular mechanism.

### Protein interaction of HERC2 with USP20 is regulated by p38 activation

**Fig. 6:**
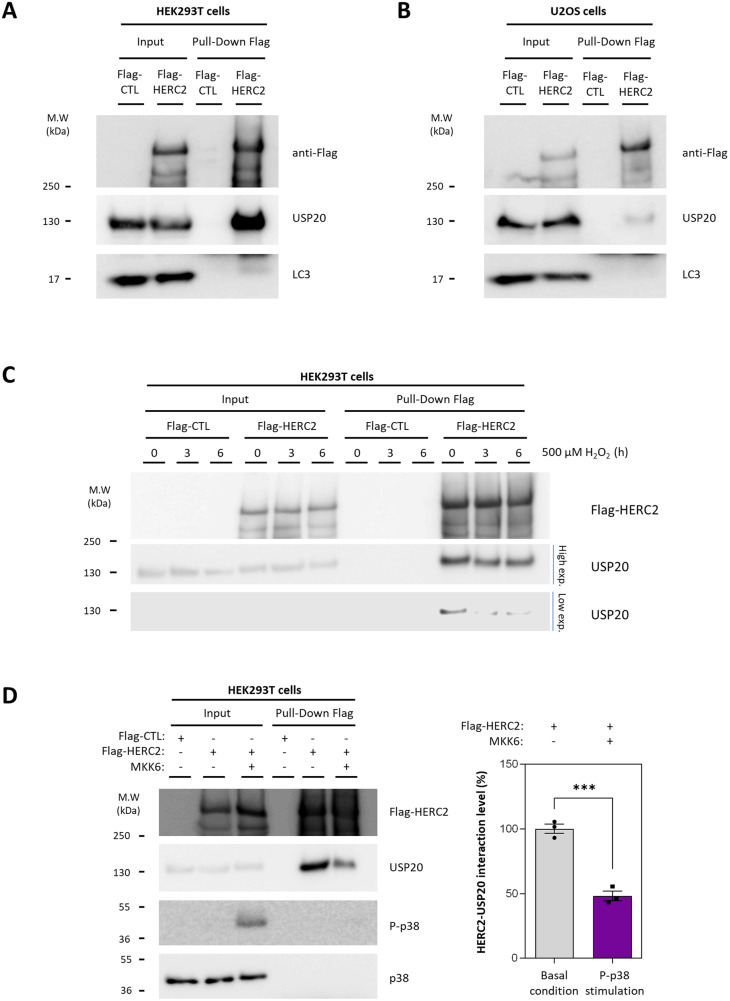
HERC2-USP20 interaction is regulated by p38 activation. HEK293T (**A**) or U2OS (**B**) cells were transfected with a control plasmid (Flag-CTL) or a plasmid encoding HERC2 protein tagged with a Flag epitope (Flag-HERC2). Cell lysates were subjected to pull-down assays using Flag-Trap agarose. Inputs and proteins retained in the resin (Pull-Down Flag) were analysed by immunoblotting with antibodies against the indicated proteins. Data are representative of three independent experiments. (**C**) HEK293T cells were transfected as in (**A**). Following transfection, the cells were exposed to 500 µM hydrogen peroxide (H_2_O_2_) for 3 or 6 h, or left untreated as a control. After the treatment, a pull-down assay was conducted as in (**A**). Immunoblot analysis was carried out to assess the levels of the indicated proteins. The data presented here are representative of two independent experiments. (**D**) HEK293T were transfected with Flag-CTL, Flag-HERC2 or Flag-HERC2 plus MKK6 plasmids. Subsequently, a pull-down assay was conducted as in (**A**). The indicated protein levels were analysed by immunoblot. The results are presented as the percentage of interaction relative to the control condition. The plot depicts the mean ± standard error of the mean (SEM). Representative results are shown for experiments that were independently repeated three times, and individual data points for each independent experimental repetition are represented as individual dots on the graph. Significance was determined using unpaired Student’s t-test, with significance level denoted as *** for *p* < 0.001.

To delve deeper into the molecular mechanisms underpinning HERC2-dependent autophagy regulation, we sought to determine whether HERC2 and USP20 proteins interact. HEK293T cells were transfected with a control plasmid (Flag-CTL), and another encoding the HERC2 protein fused with the Flag epitope (Flag-HERC2). Subsequently, we conducted a pull-down assay using a Flag-Trap resin. The results confirmed the interaction between HERC2 and USP20 proteins. No interaction was detected between HERC2 and LC3 proteins (Fig. 6A). These findings were consistent when replicated in the U2OS human cell line (Fig. 6B).

Then, we hypothesized that p38 activity might modulate the interaction between HERC2 and USP20. To investigate this, control and Flag-HERC2 transfected HEK293T cells were exposed to oxidative stress induced by H_2_O_2_ for 3 and 6 h, which is known to stimulate p38 phosphorylation and activation [26]. Following these treatments, we performed a Flag pull-down assay. After 3 and 6 h of stimulation with H_2_O_2_, the levels of USP20 interacting with HERC2 were reduced (Fig. 6C, low exposition), while the amount of pulled-down HERC2 remained unchanged (Fig. 6C). To further validate these results, HEK293T were transfected with Flag-CTL, Flag-HERC2, or a combination of Flag-HERC2 with MKK6 plasmids. Subsequent Flag pull-down assays were performed. HERC2-USP20 interaction was greatly reduced when p38 phosphorylation was induced by MKK6 overexpression (Fig. 6D). These findings provided further support for the notion that p38 activation disrupts the HERC2-USP20 interaction, a mechanism that could potentially contribute to the stabilization of USP20.

### HERC2-mediated regulation of the USP20-ULK1 axis in the HERC2-related disorder

**Fig. 7:**
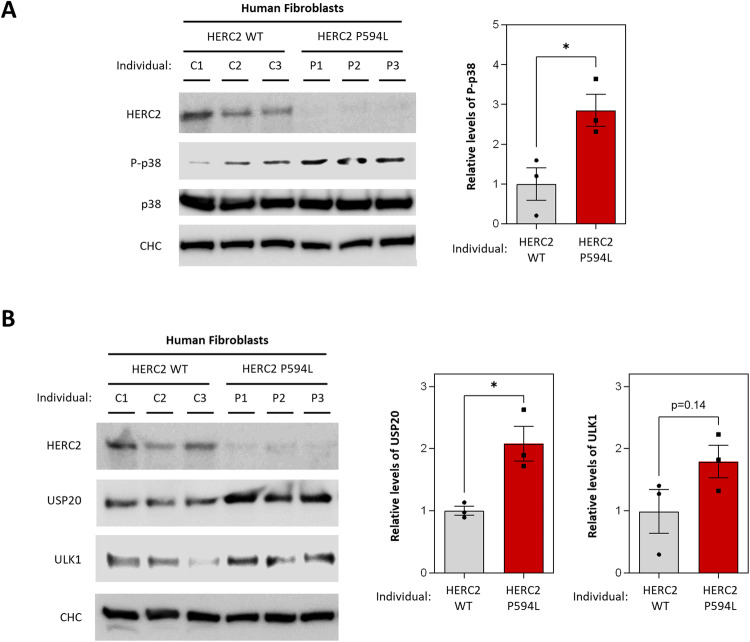
HERC2-mediated regulation of the USP20-ULK1 axis in the HERC2-related disorder. **A**, **B** Immunoblot analysis was performed on lysates derived from human skin fibroblasts, involving samples from three control individuals with the wild-type HERC2 (C1, C2 and C3) and three patients with the HERC2 P594L mutant variant (P1, P2 and P3). Band intensities corresponding to P-p38 were quantified and normalized based on total p38 protein levels (**A**); and bands corresponding to USP20 and ULK1 were quantified and subsequently normalized using clathrin heavy chain (CHC) protein levels as a loading control (**B**). The results are presented as fold changes relative to the control condition. Plots display the mean ± standard error of the mean (SEM). Representative results are shown for experiments that were independently repeated three times, and individual data points for each independent experimental repetition are represented as individual dots on the graphs. Significance was determined using unpaired Student’s t-test. Significance levels: *p < 0.05.

In a more pathophysiological context, we studied the USP20-ULK1 axis in patient-derived human fibroblasts harboring the mutant HERC2 P594L variant. These cells exhibited lower HERC2 protein levels and elevated p38 phosphorylation (Fig. 7A), as previously reported [26]. Interestingly, in this context, our results revealed a significant increase in USP20 protein levels (Fig. 7B). Regarding ULK1, we also observed a clear tendency towards increased levels (Fig. 7B).

Additionally, we conducted a fluorescence immunohistochemistry analysis of the cerebellar cortex in *Herc2*^*+/530*^ mice, which have previously been characterized by displaying approximately half-lower HERC2 protein levels [21]. Using calbindin (CaBP) as a specific marker of Purkinje cells, we evaluated the expression of USP20 and ULK1 proteins in the cerebella. We observed granular immunoreactive labeling of both proteins into three layers of the cerebellar cortex (the outer molecular layer, the middle layer of Purkinje cells, and the inner granular layer). This labeling was quantitatively more abundant in *Herc2*^*+/530*^ mice than in *Herc2*^*WT*^ mice (Supplementary Fig. 1). This upregulation likely contributes to the incremented autophagy observed in in *Herc2*^*+/530*^ mice [21]. Collectively, the results obtained in this murine model correlated with the results observed in human cells and confirmed that HERC2 deficiency is associated with the upregulation of the USP20-ULK1 axis.

## Discussion

Autophagy is a highly regulated intracellular degradation process, triggered by various signals typically converging on the activation of ULK1 [28, 29]. Ubiquitin ligases and ubiquitylation mechanisms significantly influence autophagy regulation, controlling the stability of autophagic machinery components and facilitating the recruitment of autophagy adaptors [30]. For instance, HERC1 modulates autophagy by regulating mTORC1 activity [31]. Concerning the other member of the Large HERC family, HERC2, most of available evidence regarding its involvement in autophagy has been obtained from observations in the *Herc2*^*+/530*^ mouse model [21]. These mice display a specific loss of Purkinje cells in the cerebellum, accompanied by evident signs of autophagy dysregulation. Now, we provide novel empirical evidence of a role of HERC2 as an autophagy regulator factor through the USP20-ULK1 axis, with modulation by p38 activation.

**Fig. 8:**
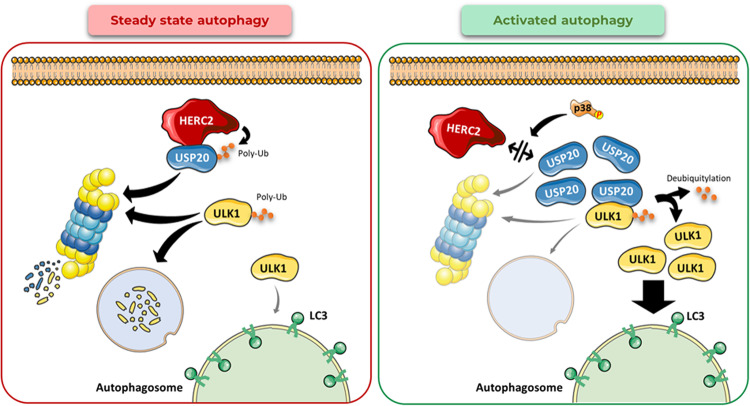
Working model of the role of HERC2 as an autophagy regulator factor. In steady-state or basal autophagy, the ubiquitin ligase HERC2 interacts with and regulates USP20 protein levels by catalyzing its ubiquitylation-dependent proteasome degradation. In this context, the protein levels of ULK1, the autophagy initiating kinase, are controlled post-translationally by ubiquitylation-mediated protein degradation (left panel). Under conditions of activated autophagy by p38 activation, HERC2-USP20 interaction is disrupted. Subsequently, USP20 protein levels are upregulated. USP20 deubiquitylates ULK1, preventing its protein degradation and hence stabilizing its protein levels. This ultimately leads to a major activation of the autophagy pathway.

In basal or steady-state autophagy conditions (Fig. 8, left panel), HERC2 interacts with USP20, potentially promoting its polyubiquitylation and subsequent proteasomal degradation. This aligns with previous research demonstrating that HERC2 interacts with and controls the protein stability of USP20 through ubiquitylation-mediated proteasomal degradation, particularly in the context of the DNA damage response pathway [23, 24]. In this report, we have demonstrated that this regulatory mechanism may also extend to the autophagy pathway. The protein levels of ULK1 are tightly controlled too. For instance, the AMBRA1-TRAF6 complex mediates K63-linked polyubiquitylation of ULK1, thereby promoting its stability [32]. On the contrary, the Cul3-KLHL20 ubiquitin ligase complex targets ULK1 for proteasomal degradation by catalyzing its K48-linked polyubiquitylation [33]. Similarly, the ubiquitin ligase NEDD4-2 ubiquitylates ULK1 marking it for proteasomal degradation [34, 35]. Additionally, ULK1 has been reported to undergo post-translational regulation by lysosomal degradation [22] (Fig. 8, left panel).

In this study, we have provided substantial evidence supporting the role of USP20 in autophagy activation through the regulation of ULK1 protein stability. Our results demonstrate that, upon HERC2 depletion and the subsequent upregulation of USP20, ULK1 protein stability is enhanced, correlating with increased autophagy. Moreover, we have shown that the increased ULK1 stability is dependent on USP20 protein levels. These observations are consistent with previously published data, which have established that USP20 deubiquitylates ULK1, safeguarding it from lysosomal degradation and enabling this kinase to effectively initiate the autophagic process [22]. Importantly, our study introduces the novel concept that HERC2 assumes the role of an upstream regulatory factor in this pathway, specifically by modulating USP20 protein levels (Fig. 8, right panel).

Further evidence supporting a role of USP20 in autophagy is derived from its subcellular localization at the endoplasmic reticulum (ER) membrane [25]. Notably, during autophagy induction, the ULK1 complex has been reported to form puncta associated with the ER. These puncta eventually develop into omegasomes, which serve as membrane extensions of the ER where some autophagosomes form [36].

Another component implicated in the regulatory mechanisms of HERC2-dependent autophagy is the MAPK p38. The role of p38 in autophagy seems multifaceted, with both positive and negative effects, possibly dependent on the stimuli and intricacies of the underlying signaling cascades [37]. Describing p38 as a positive regulator of autophagy, evidence suggests that downstream targets of p38, such as MK2 and MK3, can positively regulate starvation-induced autophagy by phosphorylating Beclin 1 [38]. Moreover, in muscle cells, p38 has been reported to induce ULK1 phosphorylation and activate autophagy [39]. Additionally, sustained p38 activation has been shown to enhance basal autophagic flux and trigger autophagosome formation through ULK1 phosphorylation [27]. However, whether p38 can regulate autophagy through directly targeting USP20 has not yet been explored.

Our study unveils that p38 activation leads to the disruption of the HERC2 and USP20 interaction. This disruption is accompanied by an increase in the protein levels of USP20 and LC3-II. These observations introduce an additional layer of USP20 regulation, suggesting that p38 may phosphorylate USP20. This phosphorylation event could trigger the dissociation of the HERC2-USP20 interaction, thereby preventing the ubiquitylation of USP20 by HERC2 and subsequently stabilizing USP20 protein levels. Consequently, elevated USP20 levels would facilitate ULK1 deubiquitylation and stabilization, ultimately promoting autophagy initiation (Fig. 8, right panel).

Deregulation of autophagy, whether up or downregulated, has been implicated in various neurodegenerative disorders, including Alzheimer’s, Huntington’s, and Parkinson’s diseases. These disorders exhibit common hallmarks such as the accumulation of autophagosomes, autolysosomes, inclusion bodies, and protein aggregates [7]. In addition, recent genetic studies have underscored the substantial involvement of autophagy in human brain development, and so in a wide range of neurodevelopmental disorders [6]. Neurons are post-mitotic and long lived cells; as such they rely on precise control of the autophagic flux to effectively eliminate dysfunctional organelles and protein aggregates. Maintaining an intricate equilibrium between autophagosome formation and autophagic degradation is of paramount importance. When the rate of autophagosome formation exceeds that of autophagic degradation, or when the later stages of the autophagic process are compromised, it can result in the accumulation of autophagic vesicles, ultimately leading to neurodegenerative processes [5, 40]. For example, loss-of-function mutations within the *TBCK* gene result in mTORC1 inhibition which triggers uncontrolled autophagy induction. These mutations cause a neurodevelopmental disorder characterized by intellectual disability. Notably, patient-derived fibroblasts in these cases exhibit an increased number of autophagosomes and an enhanced autophagic flux [41]. Similarly, we have observed an increased autophagic pathway in fibroblasts derived from individuals with the HERC2-related disorder. Additionally, previous investigations reported an elevated presence of protein aggregates in these patient-derived cells [18]. Hence, similar to the effects of the *TBCK* loss-of-function mutations, individuals with the HERC2-related disorder may experience uncontrolled autophagy induction, contributing to the accumulation of aggregates and autophagic vesicles. This could be caused by the upregulation of the USP20-ULK1 axis. Consequently, the increased autophagy might disrupt neuronal homeostasis, affecting proper neuronal development and potentially triggering neurodegenerative processes. Consistent with this hypothesis, the *Herc2*^*+/530*^ mice model exhibits cerebellar neurodegeneration, showing increased number of autophagosomes and lysosomes in their Purkinje cells [21], and thus further supporting the role of HERC2 in regulating autophagy and its implications in neurodevelopmental diseases.

Regarding the limitations of our study, we acknowledge the relatively small patient sample size employed. Biallelic *HERC2* variants associated with HERC2-related disorder are rare and have primarily been identified in Amish/Mennonite communities [17]. The rarity of these genetic disorders makes it challenging to obtain a larger cohort of patients. Additionally, the use of human fibroblasts may not fully capture the complexity of neuronal function. Consequently, further research employing different cellular models could enrich our study, complementing and enhancing the robustness of our current findings.

In summary, our findings support the role of HERC2 as an autophagy regulator factor, specifically in restricting autophagy initiation through the modulation of USP20 protein levels. Furthermore, our study indicates that this regulatory mechanism may be subject to upstream fine-tuning by p38 signaling. Taking into account all the evidence presented in this study, it suggests that targeting USP20 and/or ULK1 could potentially hold therapeutic promise for individuals affected by HERC2-related disorder and Angelman syndrome. Further investigations, including additional preclinical studies and behavioral tests in appropriate animal models, as well as in vivo experiments with specific inhibitors of these proteins, could validate this potential therapeutic approach.

## Materials and methods

### Human cell sample

Patient samples were obtained from affected individuals with HERC2 P594L mutant variant. Samples from parents and unaffected siblings were obtained and used as controls. All samples were obtained with approved informed consent in accordance with established guidelines as described in our previous study [17]. Sample size was determined based on availability.

### Cell culture

All cells were cultured in DMEM supplemented with 10% FBS, 2 mM l-glutamine, 100 U/mL penicillin, and 0.1 mg/mL streptomycin sulfate. Cells were cultured at 37 °C in a 5% CO_2_ atmosphere in a humidified incubator. HEK293T and U2O2 were obtained from ATCC (Manassas, VA, USA) and tested for mycoplasma contamination.

### Animal model

The *Herc2*^*+/530*^ C57NL/6J mice, sample size estimation, and compliance with ethical regulations were described on our previous publications [21]. Three wild-type and three *Herc2*^*+/530*^ adult mice were analyzed. No blinding was established.

### Cell treatments

Autophagic degradation in the lysosomes was inhibited by treating cells with 20 mM NH_4_Cl (MERCK # 101145) for 4 h. Autophagy was induced by exposing cells to starvation rendered by EBSS (Gibco™ # 24010043). To induce oxidative stress, cells were treated with 500 µM H_2_O_2_. All cell samples were randomized to the treatment or control groups.

### Transfections

Plasmid transfection was performed following Lipofectamine® LTX DNA Transfection Reagent Protocol (Invitrogen, # 15338100). The MKK6 plasmid was a gift from Dr. Roger Davis (Addgene plasmid # 13518; http://n2t.net/addgene:13518; RRID:Addgene_13518) [42]. The Flag-HERC2 plasmid was a gift from Dr. David Chan (Addgene plasmid # 55613; http://n2t.net/addgene:55613; RRID:Addgene_55613) [43].

For gene knockdown, silencing RNAs (siRNAs) were transfected using the calcium phosphate method described elsewhere [44]. Custom double-stranded siRNA oligonucleotides were obtained from GeneCust (Boynes, France). Sequences available in Supplementary Table 1.

### Protein analysis

Protein extraction and determination of specific protein levels was performed as previously described [26]. In brief, cells were washed twice in ice-cold phosphate-buffer saline after discarding the media. Cells were lysed by scrapping after adding NP40 lysis buffer (50 mM Tris–HCl, 150 mM NaCl, and 0.5% NP40 detergent, pH 7.5). This buffer was supplemented with protease and phosphatase inhibitors to avoid degradation of proteins and the removing of phosphorylation marks (50 mM β-glicerophosphate, 50 mM sodium fluoride (NaF), 1 mM sodium vanadate, 1 mM phenylmethylsulfonyl fluoride (PMSF), 5 µg/mL leupeptin, 5 µg/mL aprotinin, 1 µg/mL pepstatin A, 100 µg/mL benzamidine, 1 µM E-64). Lysates were maintained on ice under agitation for 20 min, and then centrifuged at 13,000 × *g* at 4 °C for 10 min. Supernatants were collected before protein analysis using the Tris–Acetate PAGE system [45]. For standard gradient gels, a 3–15% acrylamide gradient was employed. The separation of LC3-I and LC3-II proteins was achieved using a 3–17.5% gradient. A list of the antibodies used in this study is provided in Supplementary Table 2.

### Monitoring of autophagy

Autophagy was measured by immunoblotting based on the quantification of the LC3-II protein using an anti-LC3B antibody (Santa Cruz Biotechnology # sc-376404). The difference in the amount of LC3-II between cells treated with or without lysosome inhibitors represented the level of autophagic flux, as previously described [46].

### Subcellular fractionation

Cells were permeabilized with 0.015% digitonin and fractionated into cytosol and membrane fractions as described elsewhere [25].

### Pull-down assays

For the pull-down assays, cells were lysed as previously described [26]. Samples were incubated with 5 μL of DYKDDDDK Fab-Trap™ Agarose (ChromoTek # ffa) for 2 h at 4 °C with gentle rotation. Pellets were washed five times and analyzed by immunoblot.

### Immunohistochemistry

Immunohistochemistry analysis of the cerebellum was performed as previously described [21].

### Statistical analysis

The plotted values represent the means with standard error of the mean (±SEM) from a minimum of three independent experiments. Each data point from independent repetitions is individually displayed on the respective graph. Significance was determined through either the Student t-test or either one-way or two-way analysis of variance (ANOVA) with subsequent correction for multiple comparisons using Tukey’s test. Levels of significance are denoted as follows: *, **, or *** for *p*-values of <0.05, <0.01, or <0.001, respectively. All figures were generated, and statistical analysis were conducted, using GraphPad Prism version 8.4.3 for Windows (GraphPad Software, San Diego, CA, USA), available at www.graphpad.com.

### Supplementary information

RawData

Supplementary materials

## Data Availability

The data that support the findings of this study are available from the corresponding author upon reasonable request.
