# Supplementary material for: Autophagy dysregulation via the USP20-ULK1 axis in the HERC2-related neurodevelopmental disorder
Source: Cell Death Discov. 2024 Apr 3;10:163. doi: 10.1038/s41420-024-01931-6 (PMC10991529; doi:10.1038/s41420-024-01931-6)

Figure 1

A

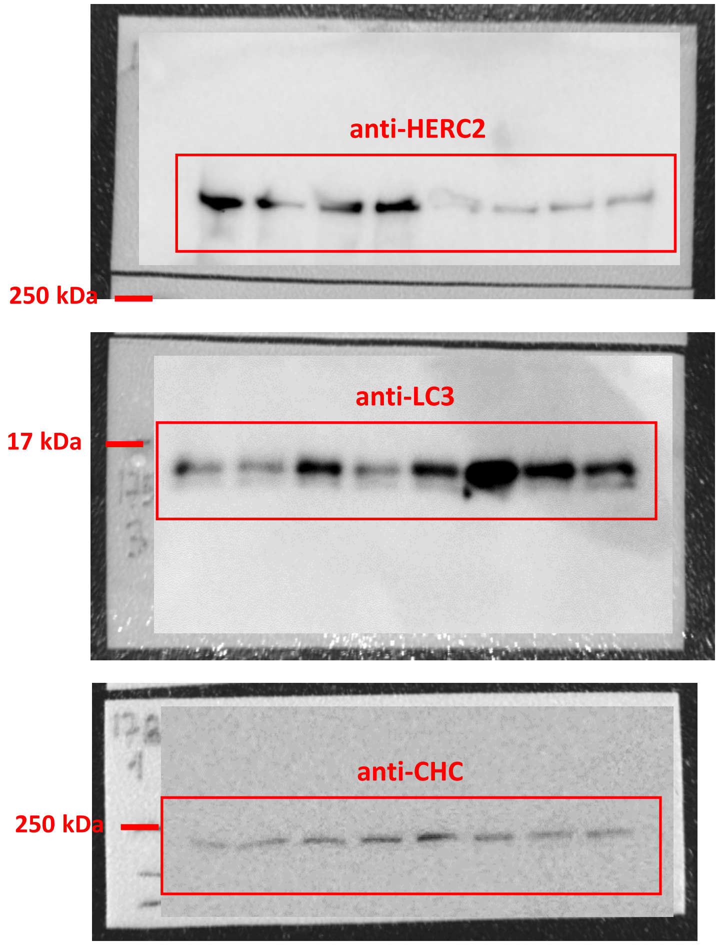

Figure 1

B

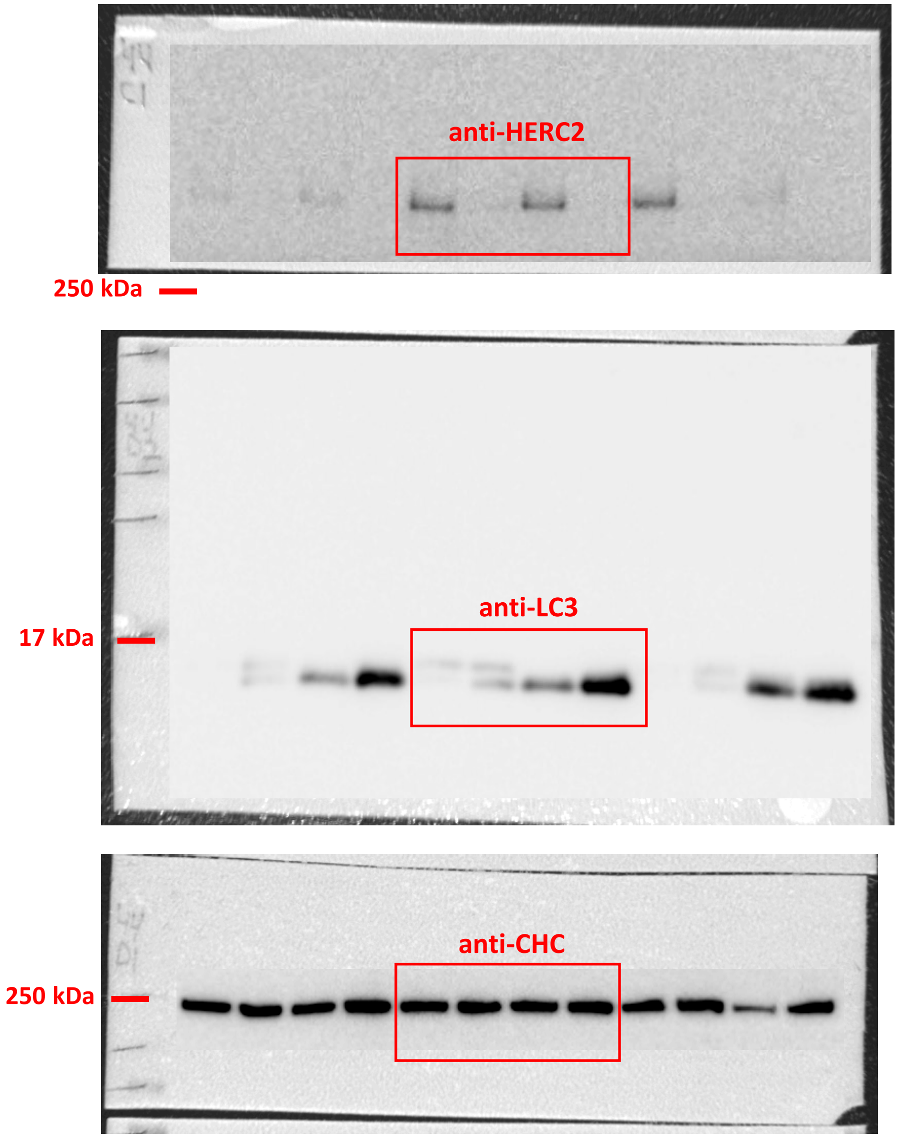

Figure 1

C

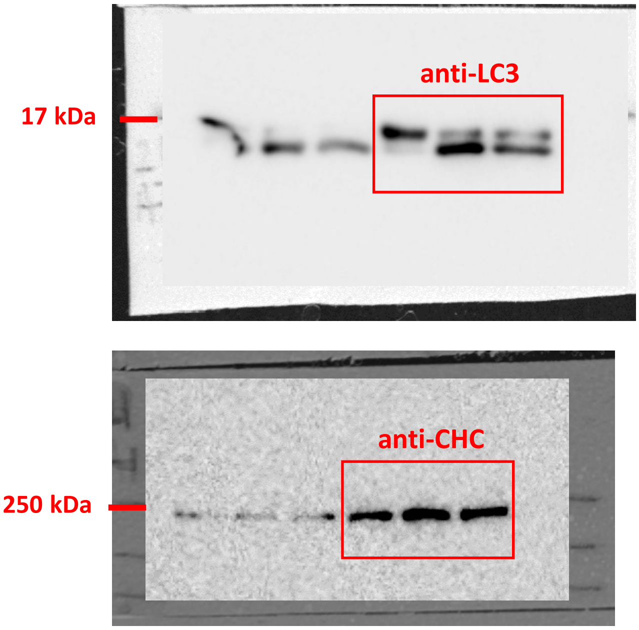

anti-LC3
17 kDa
anti-CHC
250 kDa

D

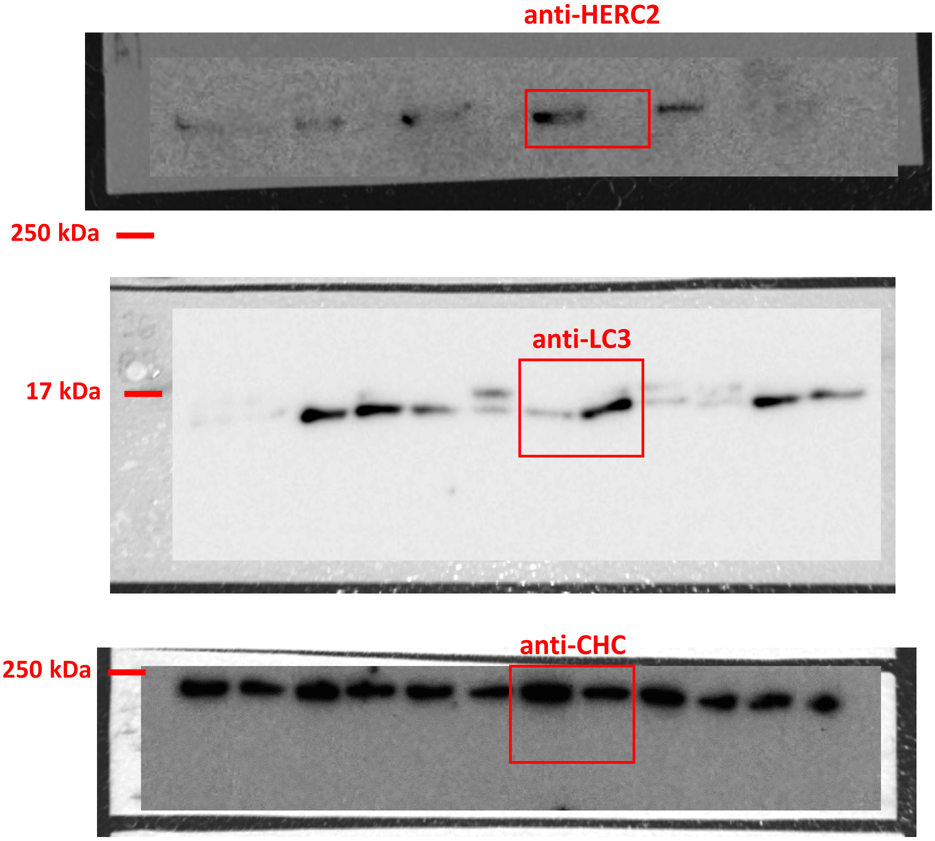

anti-HERC2
250 kDa
anti-LC3
17 kDa
anti-CHC
250 kDa

Figure 2

A

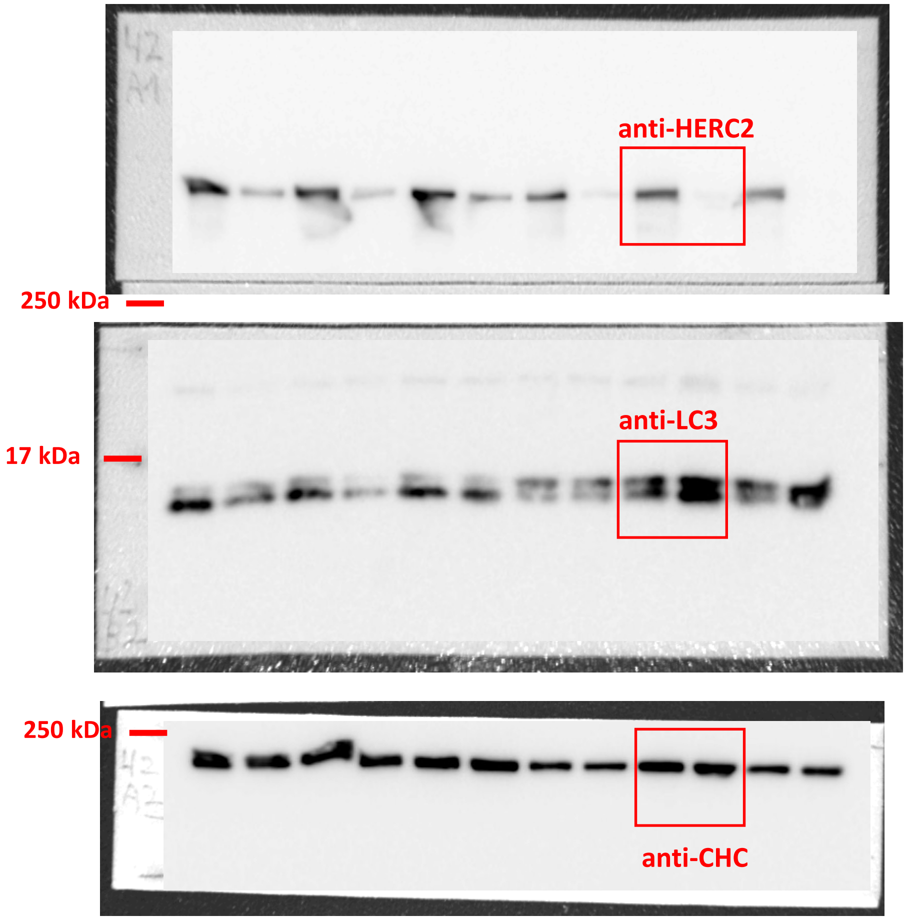

Figure 2

B

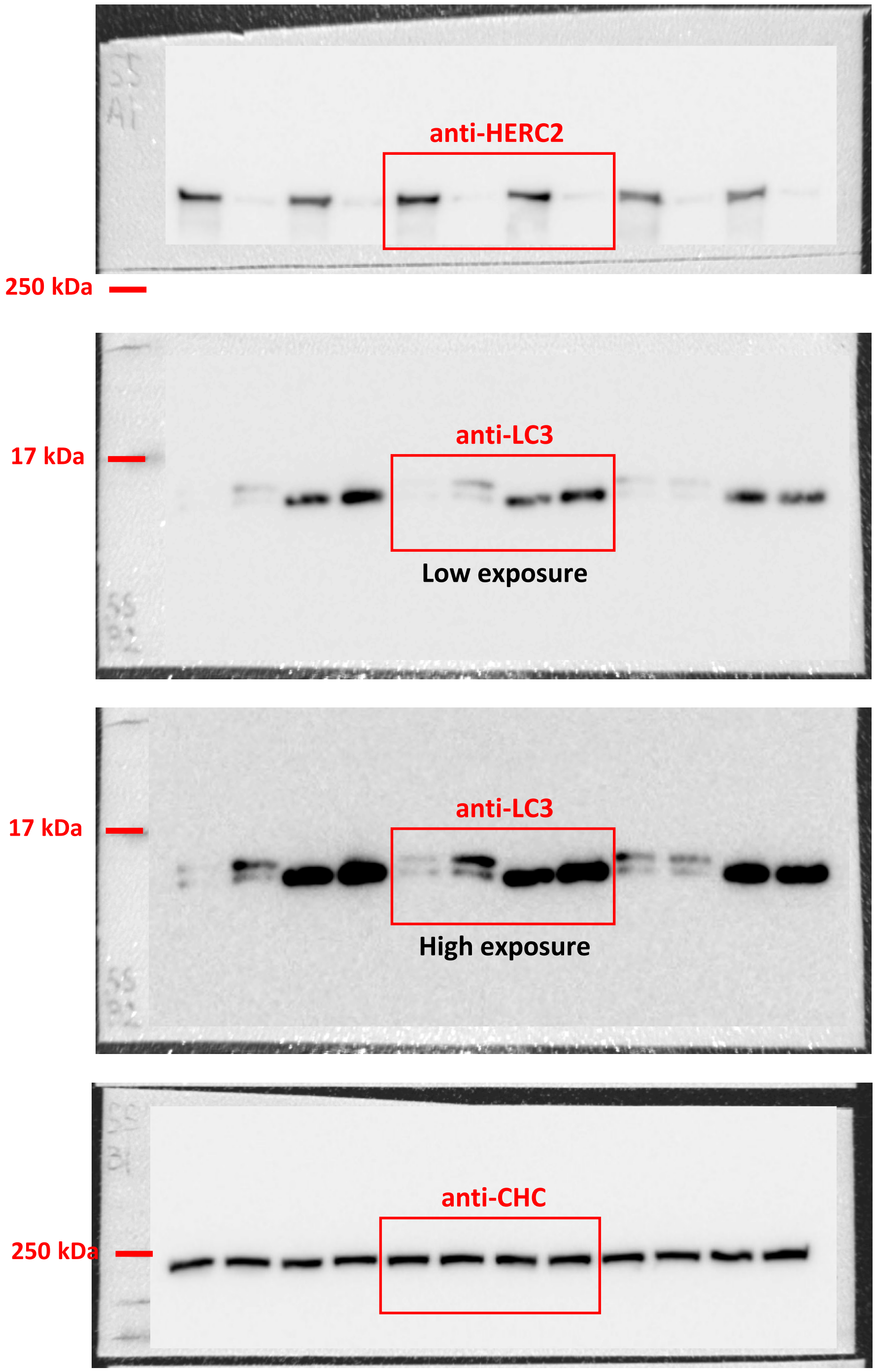
anti-HERC2
250 kDa
17 kDa
anti-LC3
Low exposure
17 kDa
anti-LC3
High exposure
anti-CHC
250 kDa

Figure 3

B

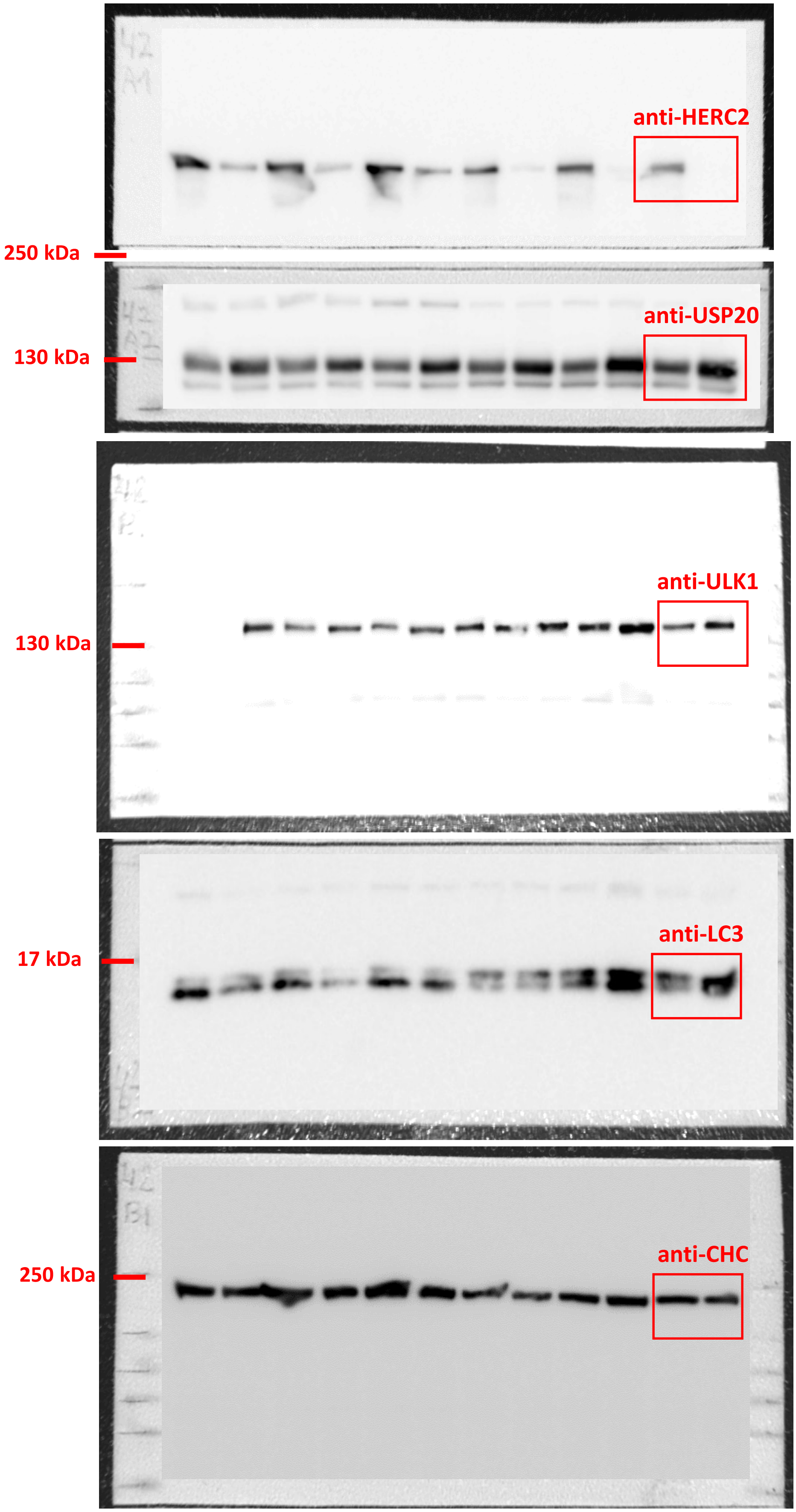

anti-HERC2
250 kDa
anti-USP20
130 kDa
anti-ULK1
130 kDa
anti-LC3
17 kDa
anti-CHC
250 kDa

Figure 3

C

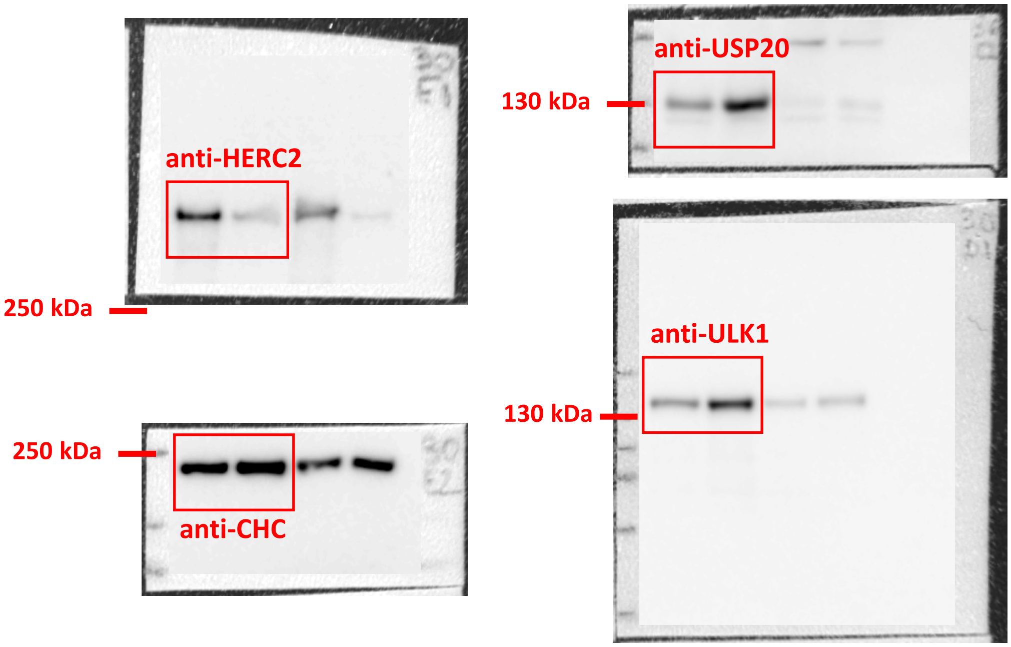
anti-HERC2
250 kDa
250 kDa
anti-CHC
anti-USP20
130 kDa
anti-ULK1
130 kDa

D

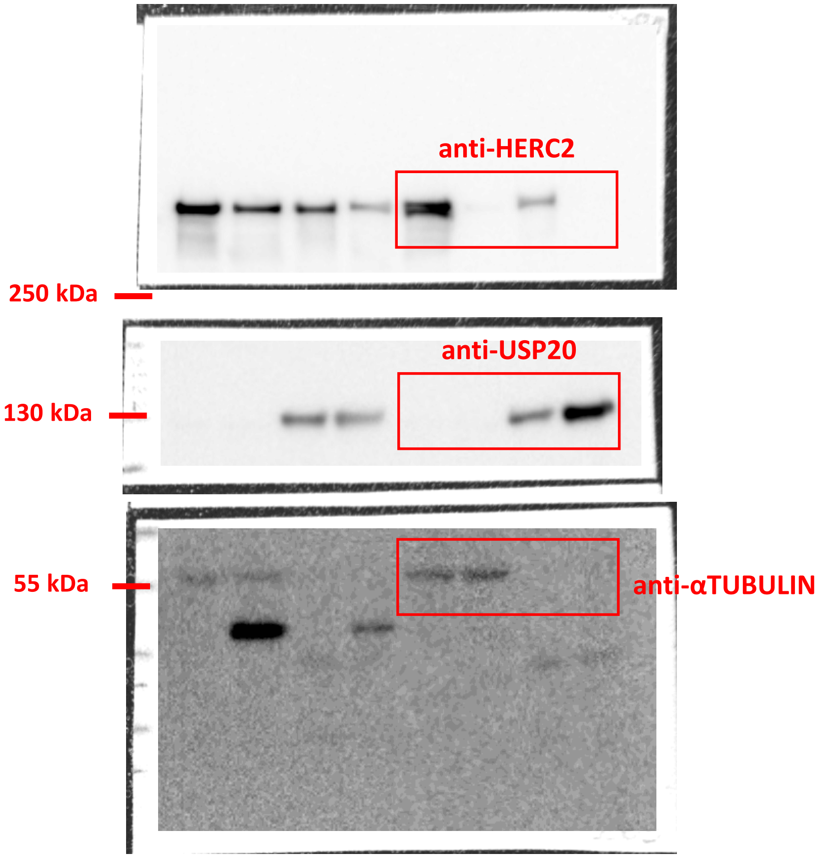
anti-HERC2
250 kDa
anti-USP20
130 kDa
55 kDa
anti-αTUBULIN

Figure 4

A

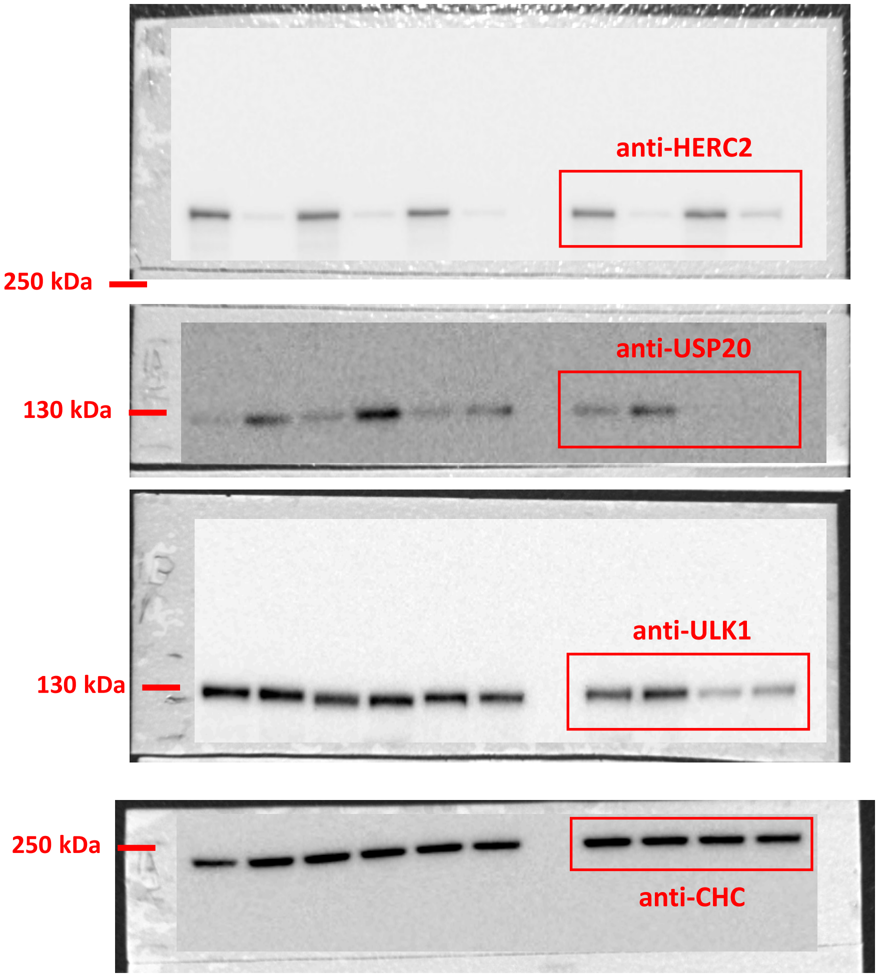
anti-HERC2
250 kDa
anti-USP20
130 kDa
anti-ULK1
130 kDa
250 kDa
anti-CHC

Figure 4

B

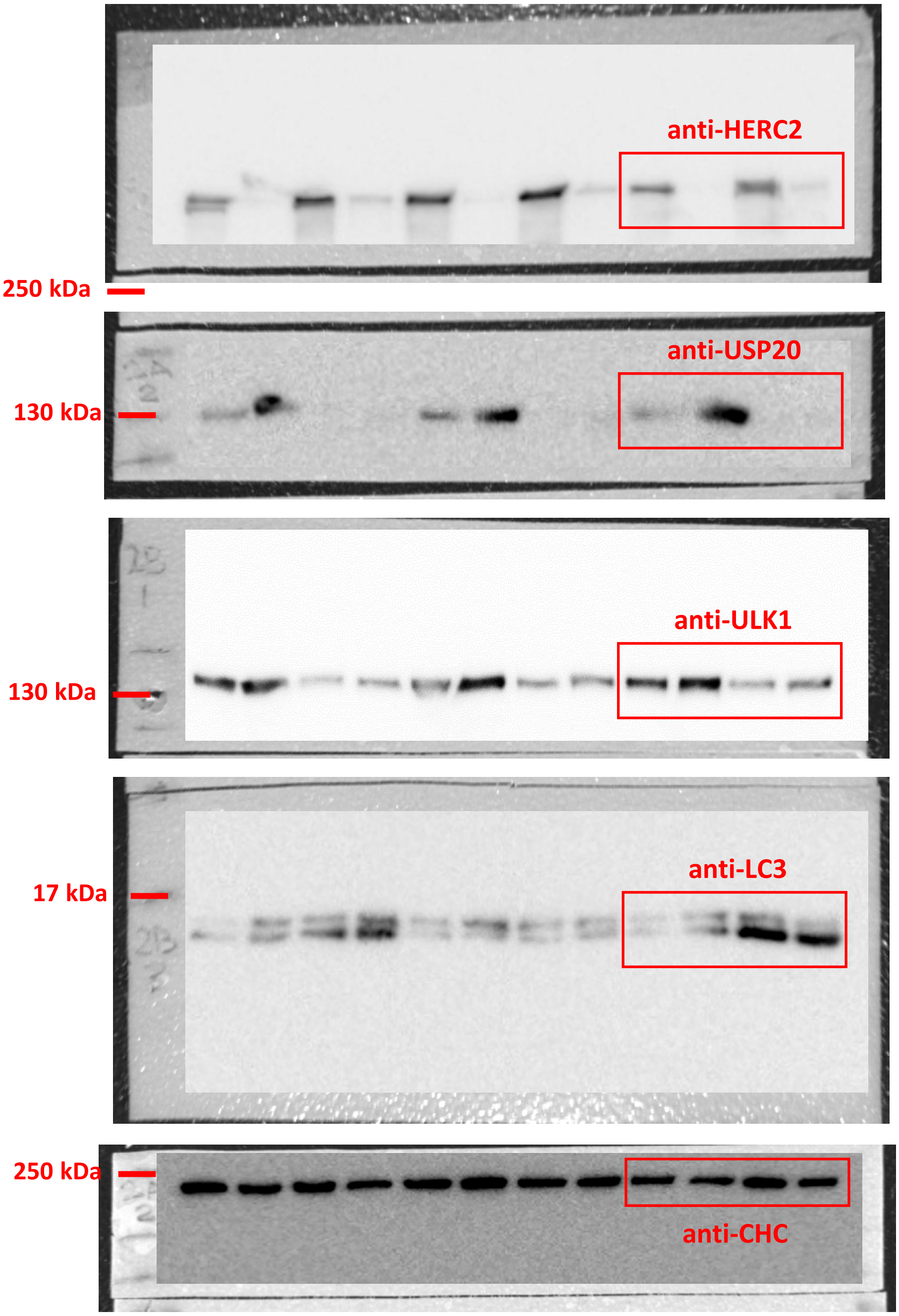
anti-HERC2
250 kDa
anti-USP20
130 kDa
anti-ULK1
130 kDa
anti-LC3
17 kDa
250 kDa
anti-CHC

Figure 5

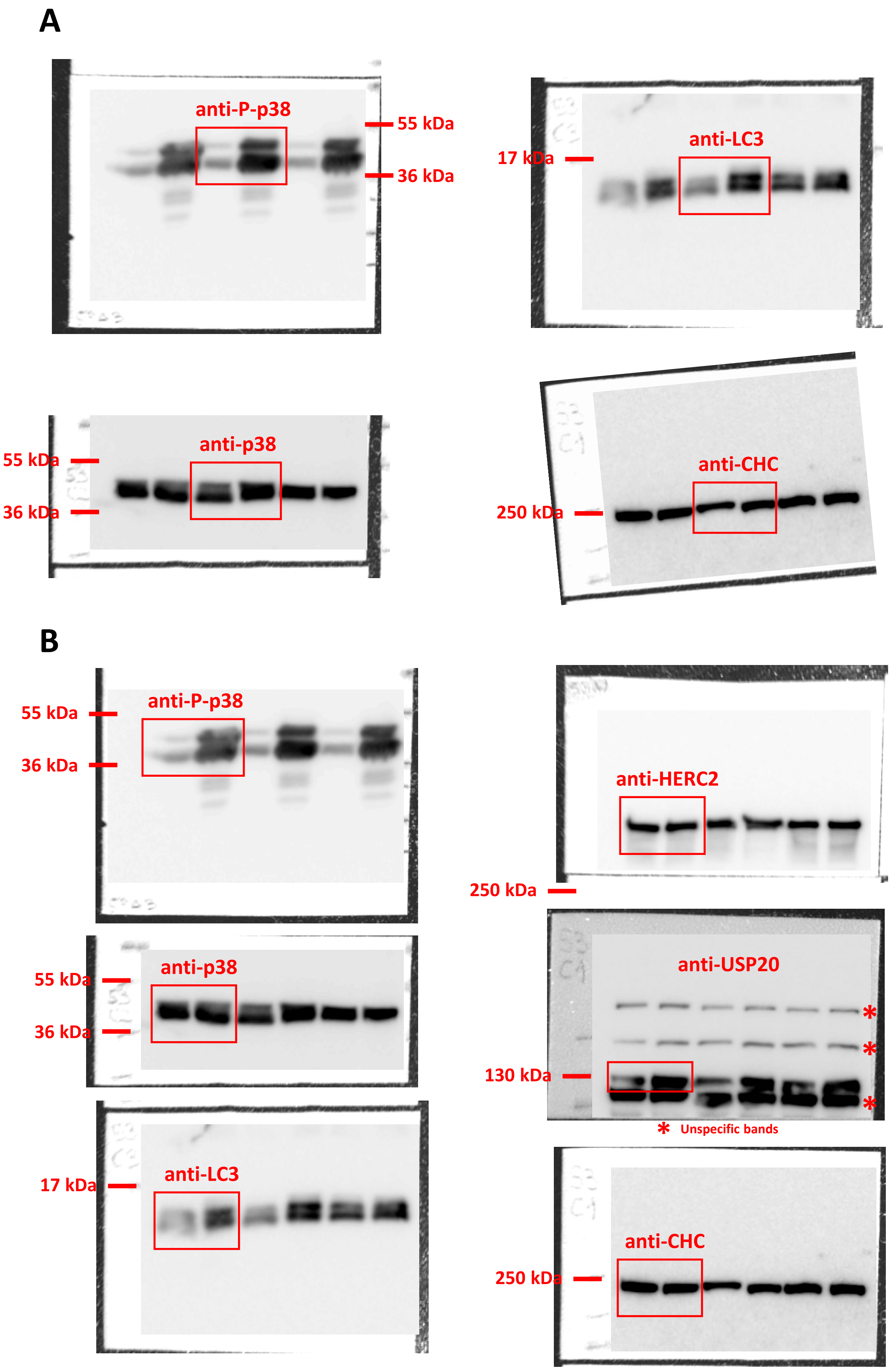
A
anti-P-p38
55 kDa
36 kDa
anti-LC3
17 kDa
anti-p38
55 kDa
36 kDa
anti-CHC
250 kDa
B
anti-P-p38
55 kDa
36 kDa
anti-HERC2
250 kDa
anti-p38
55 kDa
36 kDa
anti-USP20
130 kDa
* Unspecific bands
anti-LC3
17 kDa
anti-CHC
250 kDa

Figure 6

A

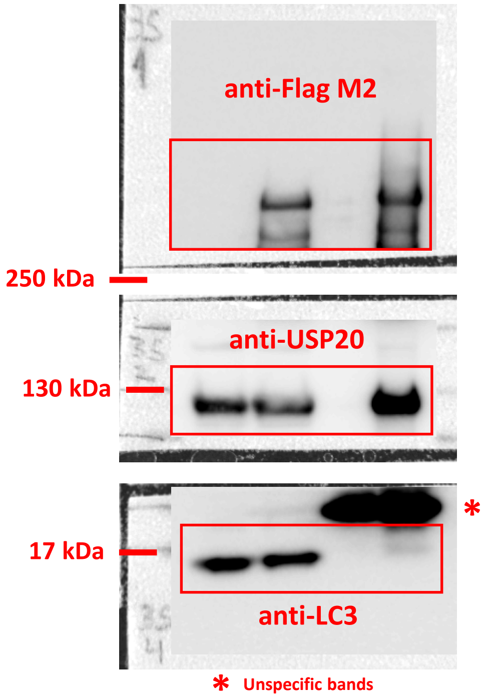
anti-Flag M2
250 kDa
anti-USP20
130 kDa
*
17 kDa
anti-LC3
* Unspecific bands

B

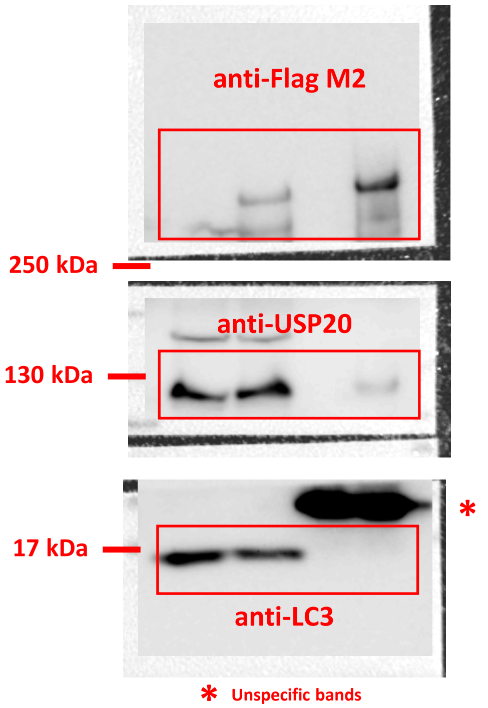
anti-Flag M2
250 kDa
anti-USP20
130 kDa
*
17 kDa
anti-LC3
* Unspecific bands

D

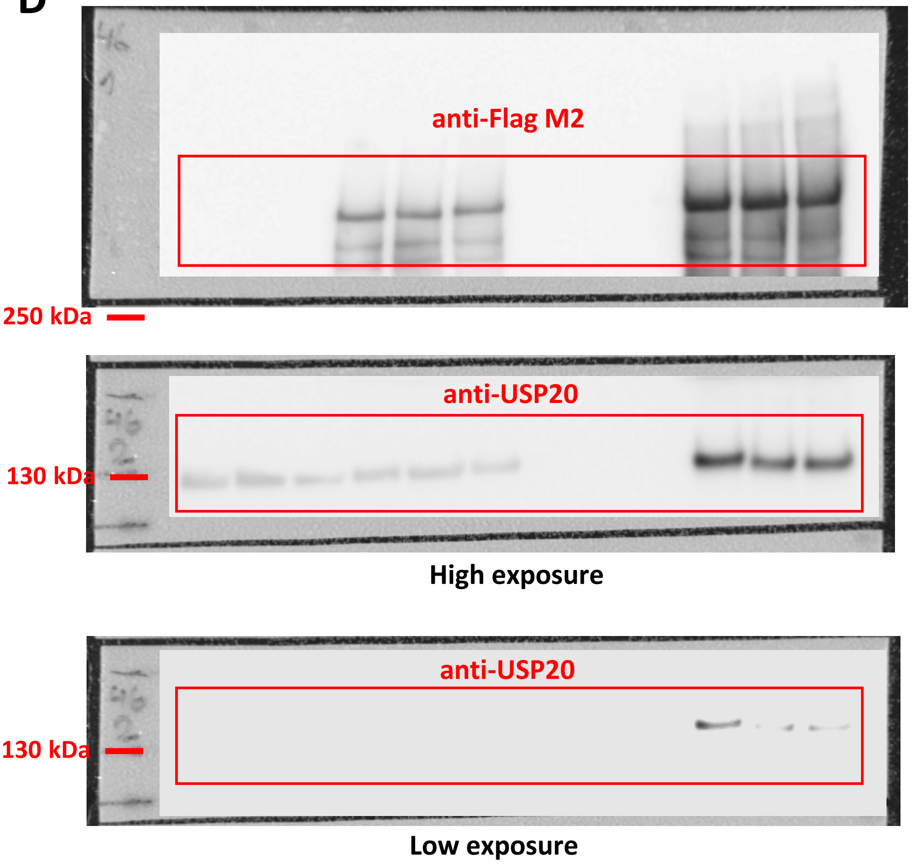
anti-Flag M2
250 kDa
anti-USP20
130 kDa
High exposure
anti-USP20
130 kDa
Low exposure

Figure 6

E

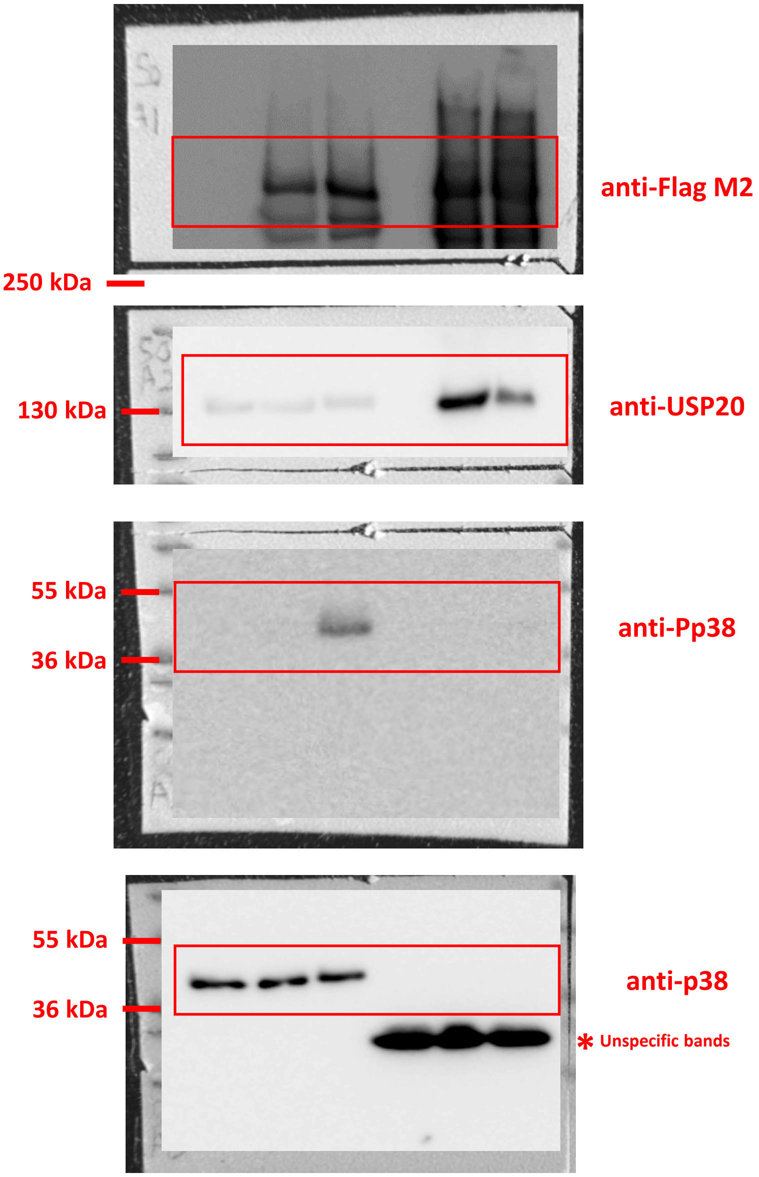

anti-Flag M2
250 kDa
130 kDa
anti-USP20
55 kDa
36 kDa
anti-Pp38
55 kDa
36 kDa
anti-p38
* Unspecific bands

Figure 7

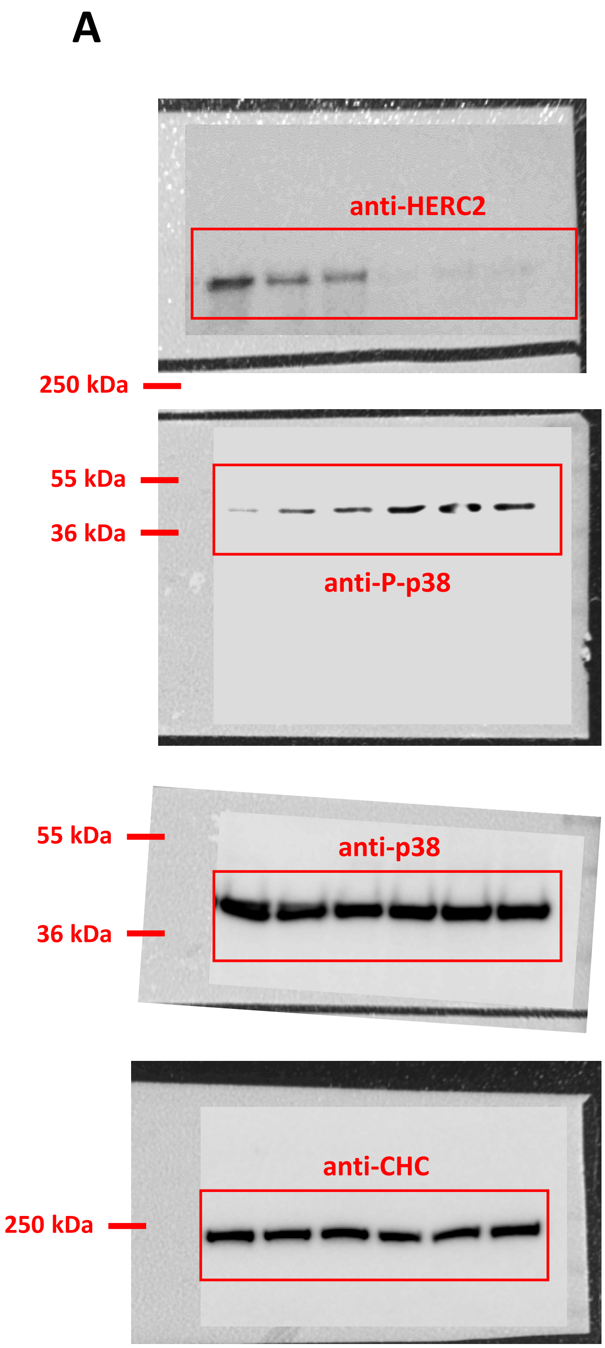
A
anti-HERC2
250 kDa
55 kDa
36 kDa
anti-P-p38
55 kDa
anti-p38
36 kDa
anti-CHC
250 kDa

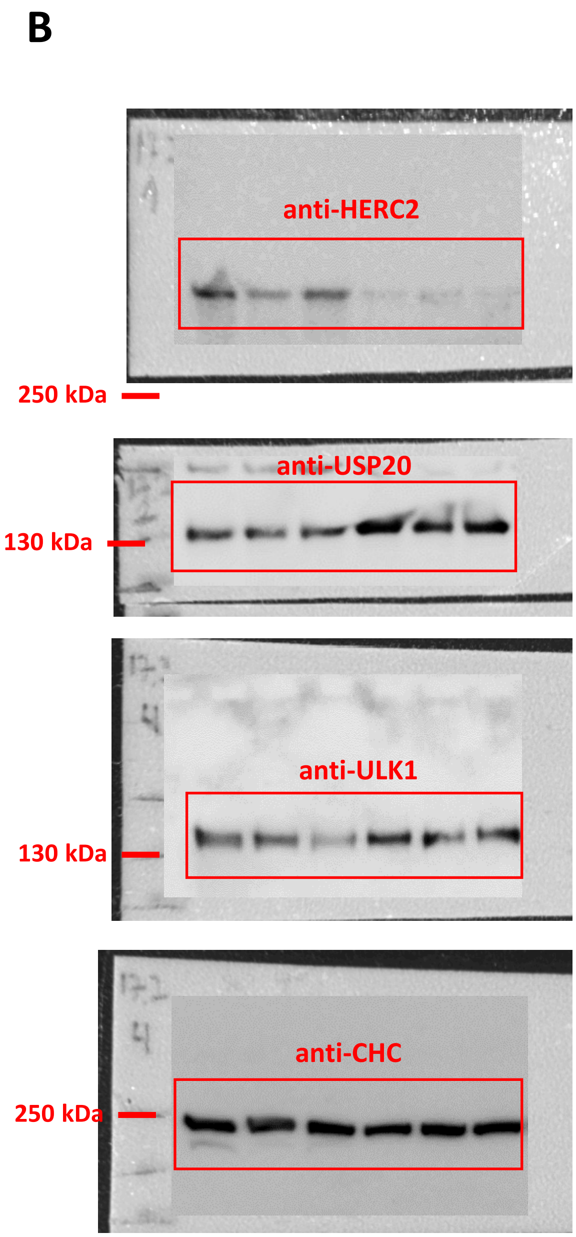
B
anti-HERC2
250 kDa
anti-USP20
130 kDa
anti-ULK1
130 kDa
anti-CHC
250 kDa

Supplementary figure 1

A

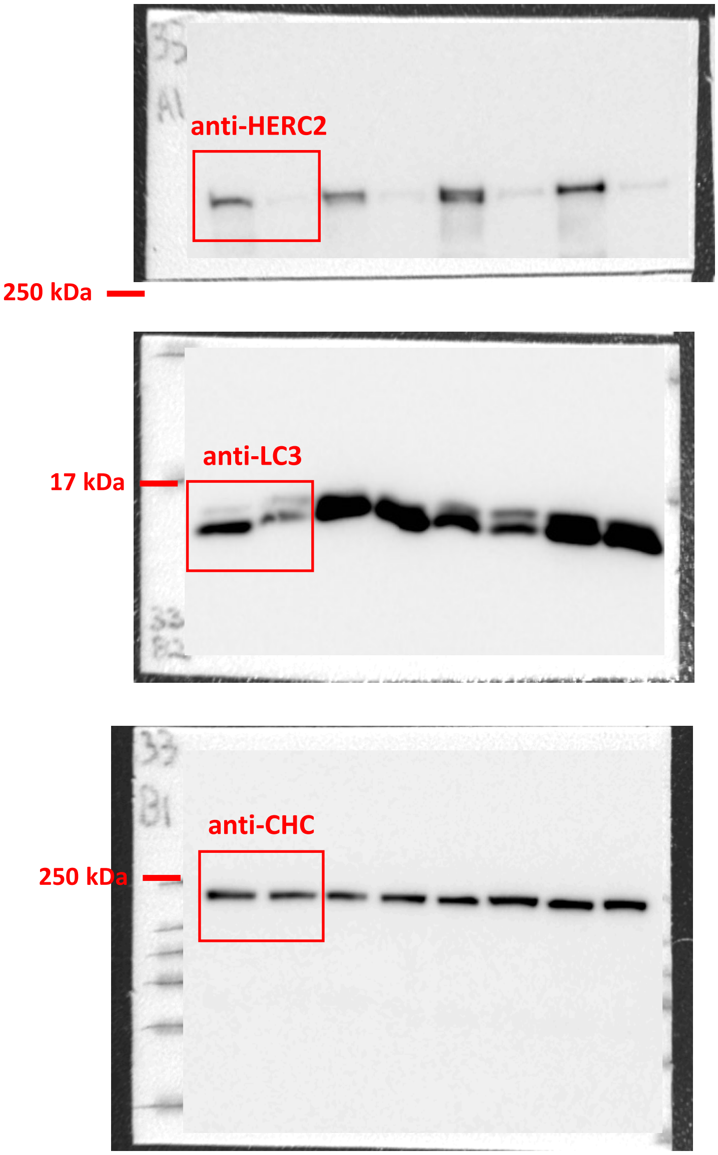

Supplementary figure 1

B

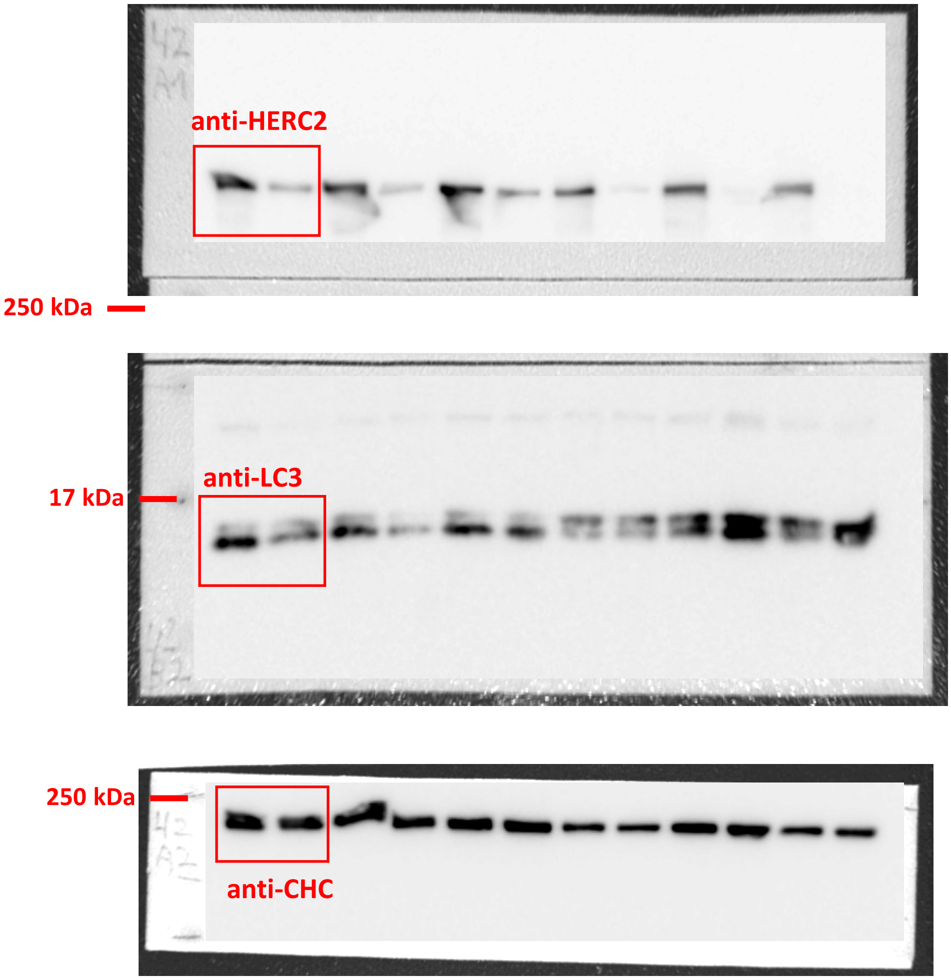

Supplementary figure 1

C

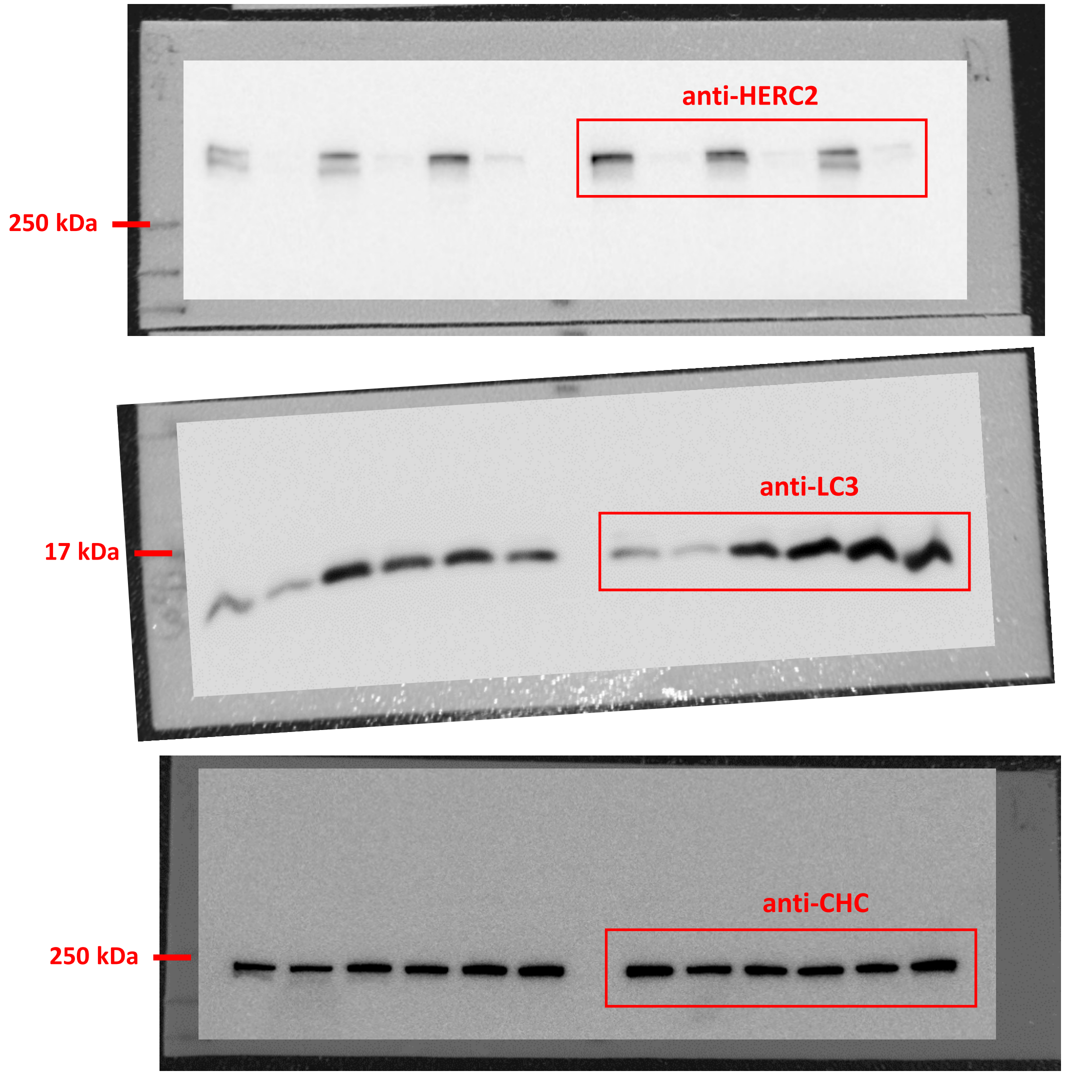

D

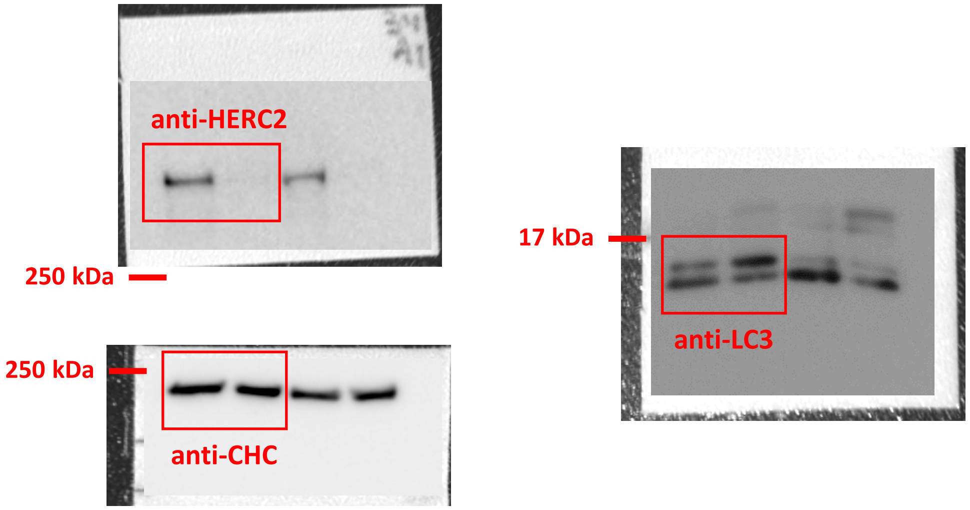

Supplement: Supplementary file 1 — RawData [file 41420_2024_1931_MOESM1_ESM.pdf]
