# Supplementary material for: Autophagy dysregulation via the USP20-ULK1 axis in the HERC2-related neurodevelopmental disorder
Source: Cell Death Discov. 2024 Apr 3;10:163. doi: 10.1038/s41420-024-01931-6 (PMC10991529; doi:10.1038/s41420-024-01931-6)

SUPPLEMENTARY MATERIALS

This supplementary file provides further methodology information, including the siRNA sequences and antibodies used in this study. Additionally, Supplementary Figure 1 is included to further support the findings presented in the main manuscript.

Supplementary Table 1. List of siRNAs and their specific forward sequences.

siRNA	Forward sequence
**Negative control (NC)**	5′-UUCUCCGAACGUGUCACGU**TT**-3′
**HERC2**	5′-GACUGUAGCCAGAUUGAAA**TT**-3′
**USP20**	5′-GGACAATGATGCTCACCTA**TT**-3′

Supplementary Table 2. List of antibodies.

Antibody	Reference
**anti-HERC2**	BD Biosciences # 612366
**anti-LC3B (G-9)**	Santa Cruz Biotechnology # sc-376404
**anti-Clathrin Heavy Chain (TD.1)**	Santa Cruz Biotechnology # sc-12734
**anti-USP20 polyclonal**	Proteintech # 17491-1-AP
**anti-ULK1 (D8H5)**	Cell Signaling # 8054
**anti-α TUBULIN (B-7)**	Santa Cruz Biotechnology # sc-5286
**anti-phospho-p38**	Cell signaling # 9211
**anti-p38**	Santa Cruz Biotechnology # sc-535
**anti-Flag M2**	Sigma-Aldrich # F3165
**anti-Calbindin-D-28K**	Sigma-Aldrich # SAB4200543
**secondary antibodies**	Invitrogen

Supplementary Figure 1. Expression of USP20 and ULK1 proteins in the cerebella. Confocal laser microphotographs of cerebellar coronal sections of Herc2WT mice (**A**, **C**) and Herc2+/530 mice (**B**, **D**). Immunoreactive labeling of USP20 and ULK1 proteins into the three layers: the outer molecular (mol) layer, the middle layer of Purkinje cells (Pc) and the inner granular (g) layer. Quantitative analysis of the number of immunoreactive granules for USP20 and ULK1 proteins (**E**, **F**). CaBP, calbindin; DAPI (4′,6-diamidino-2-phenylindole), a blue-fluorescent DNA stain. Bars = 50 µm. The plots in (**E**, **F**) display the mean ± standard error of the mean (SEM). Significance was determined using unpaired Student's t-test. Significance level: *** $p < 0.001$.

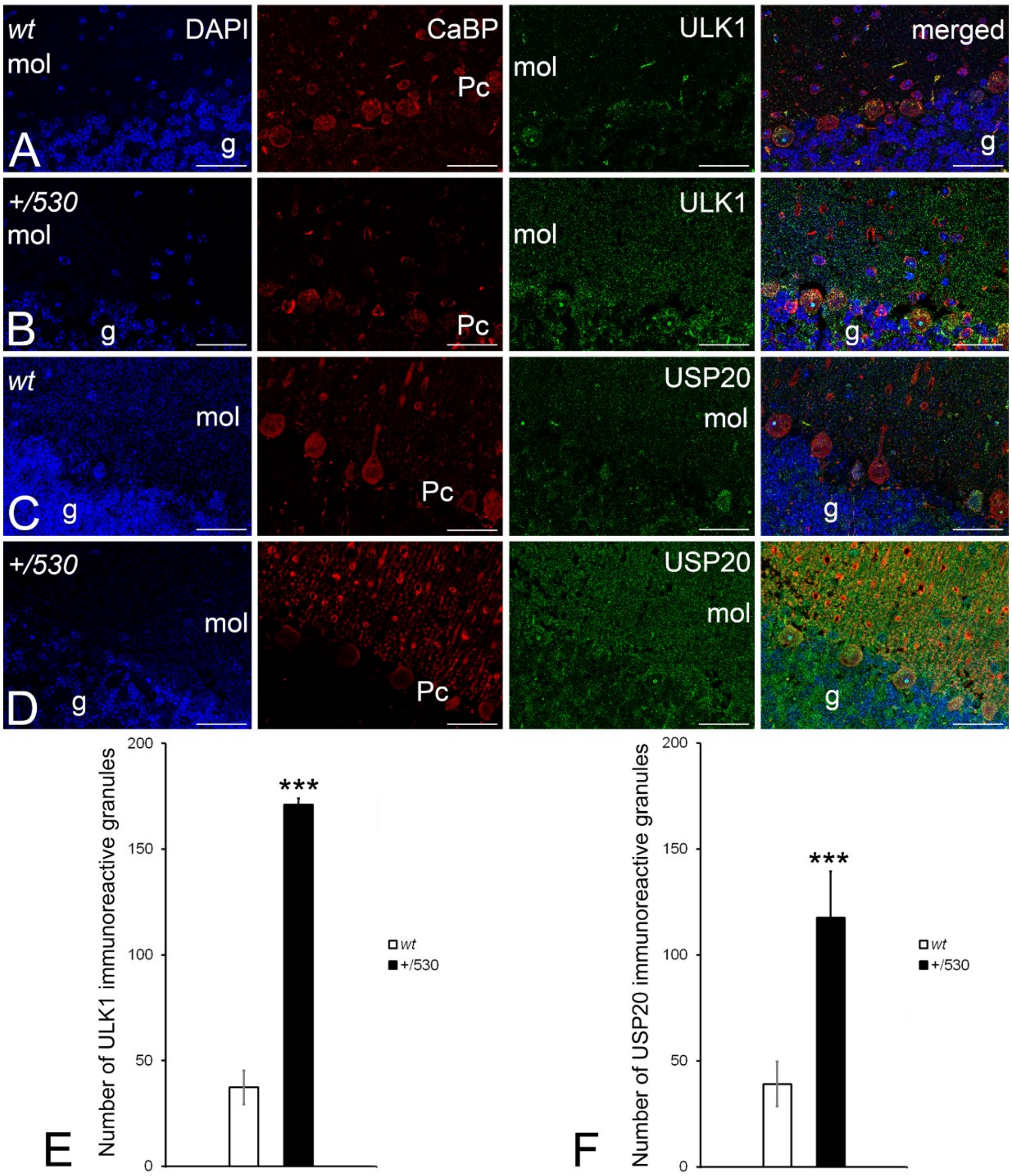

Supplement: Supplementary file 2 — Supplementary materials [file 41420_2024_1931_MOESM2_ESM.pdf]
